# Supplementary material for: Nonlinear expression patterns and multiple shifts in gene network interactions underlie robust phenotypic change in Drosophila melanogaster selected for night sleep duration
Source: PLoS Comput Biol. 2023 Aug 10;19(8):e1011389. doi: 10.1371/journal.pcbi.1011389 (PMC10443883; doi:10.1371/journal.pcbi.1011389)
Supplement: S1 Fig — PCA on matrix of normalized expression data shows complete separation of sexes along the first component, which explains 65% of the variance in the data. (PDF) [file pcbi.1011389.s001.pdf]

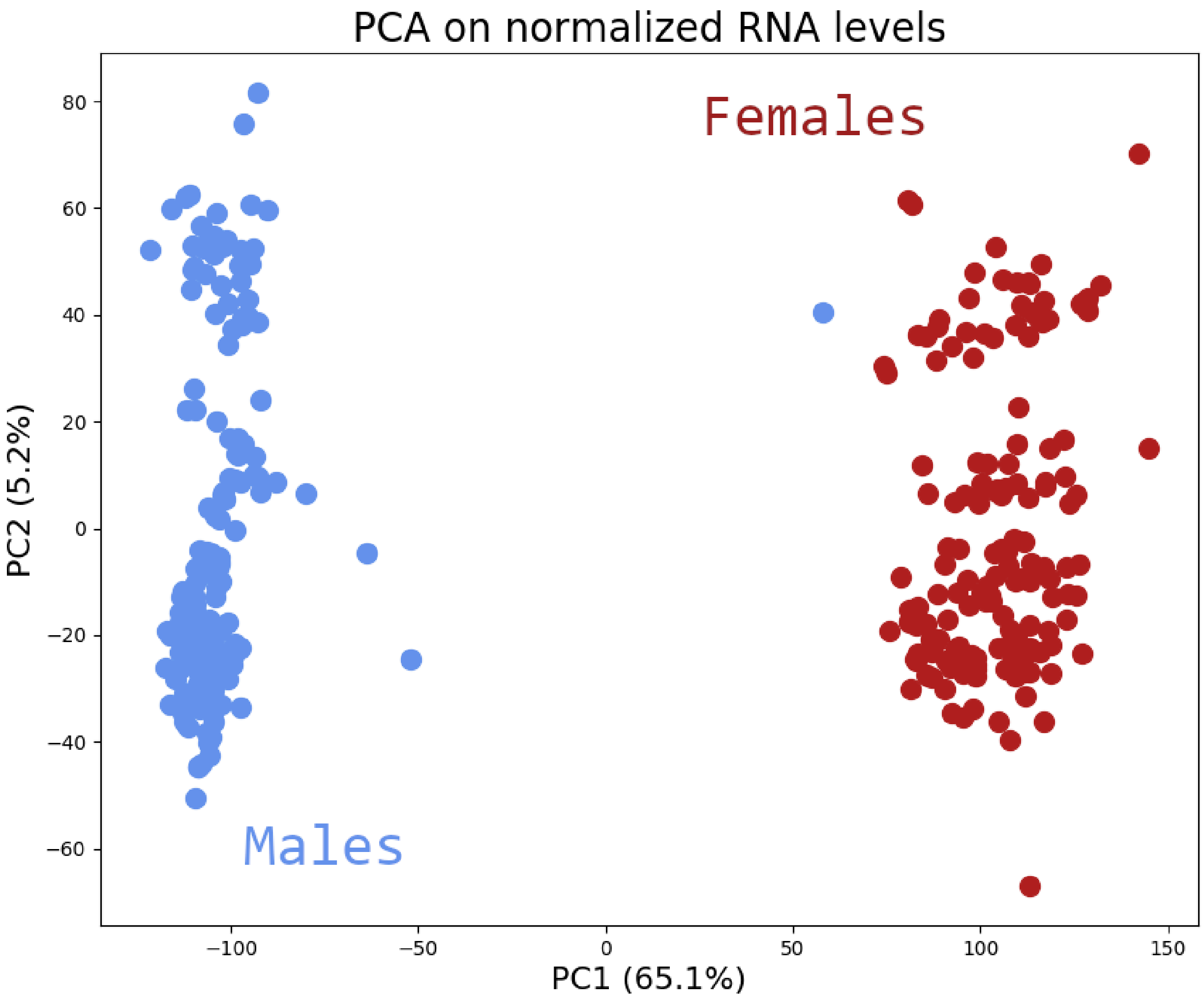

S1 Fig. Principal Component Analysis (PCA).
PCA on matrix of normalized expression data shows complete separation of sexes along the first component, which explains 65\% of the variance in the data.
