# Supplementary material for: Nonlinear expression patterns and multiple shifts in gene network interactions underlie robust phenotypic change in Drosophila melanogaster selected for night sleep duration
Source: PLoS Comput Biol. 2023 Aug 10;19(8):e1011389. doi: 10.1371/journal.pcbi.1011389 (PMC10443883; doi:10.1371/journal.pcbi.1011389)
Supplement: S2 Fig — A, day bout number; B, day bout number coefficient of environmental variation (CVE); C, day sleep; D, day sleep CVE; E, night bout number; F, night bout number CVE; G, night sleep; H, night sleep CVE; I, waking activity; J, waking activity CVE; K, sleep latency; L, sleep latency CVE; M, day average bout length; N, day average bout length CVE; O, night average bout length; P, night average bout length CVE; Q, night sleep standard deviation. Light green, Replicate 1 long-sleeper population; Dark green, Replicate 2 long-sleeper population; Orange, Replicate 1 short-sleeper population; Red, Replicate 2 short-sleeper population; Gray, Replicate 1 control population; Black, Replicate 2 control population. CVE, coefficient of environmental variation. (PDF) [file pcbi.1011389.s002.pdf]

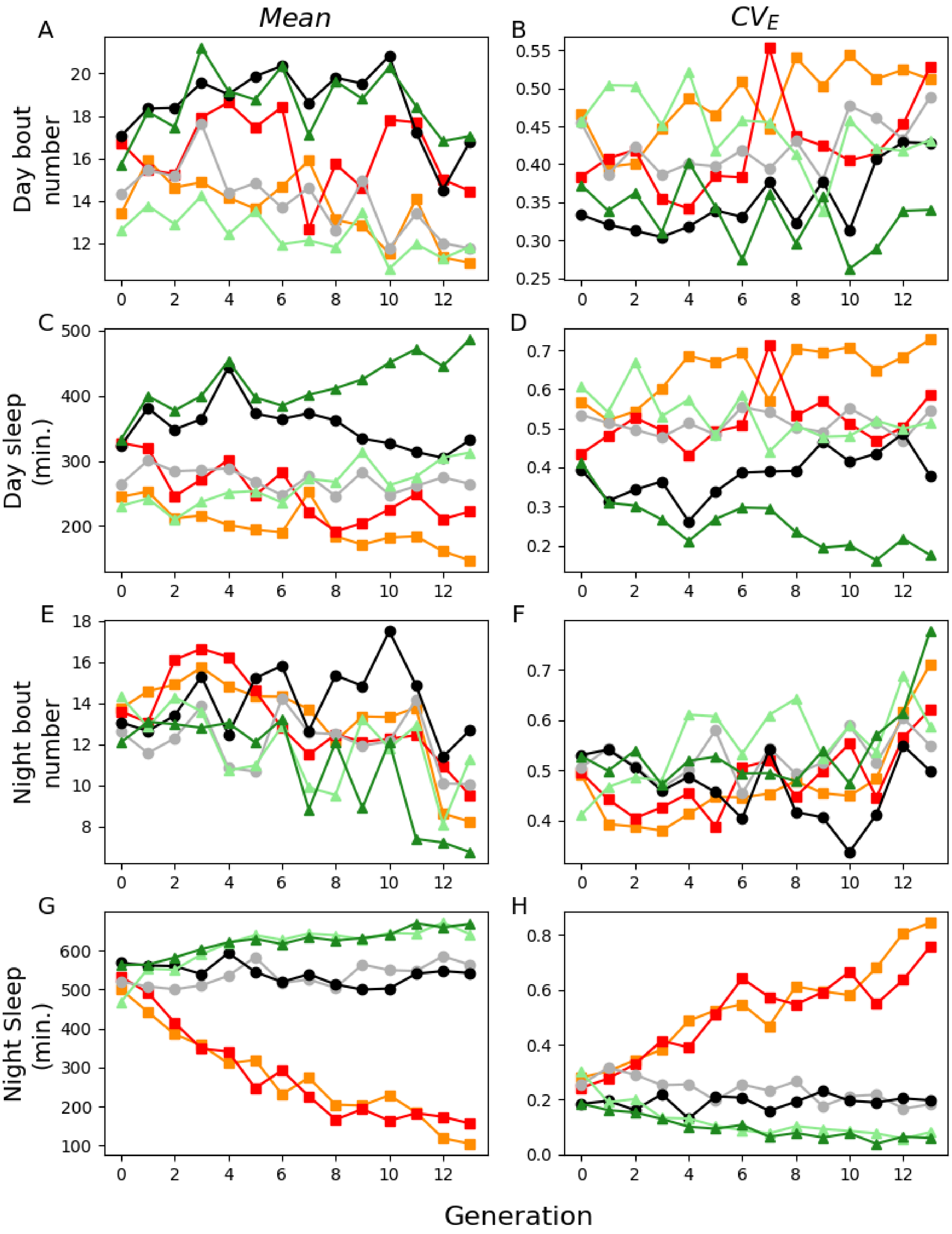

Mean
CV_E
A
B
C
D
E
F
G
H
Day bout number
Day sleep (min.)
Night bout number
Night Sleep (min.)
Generation

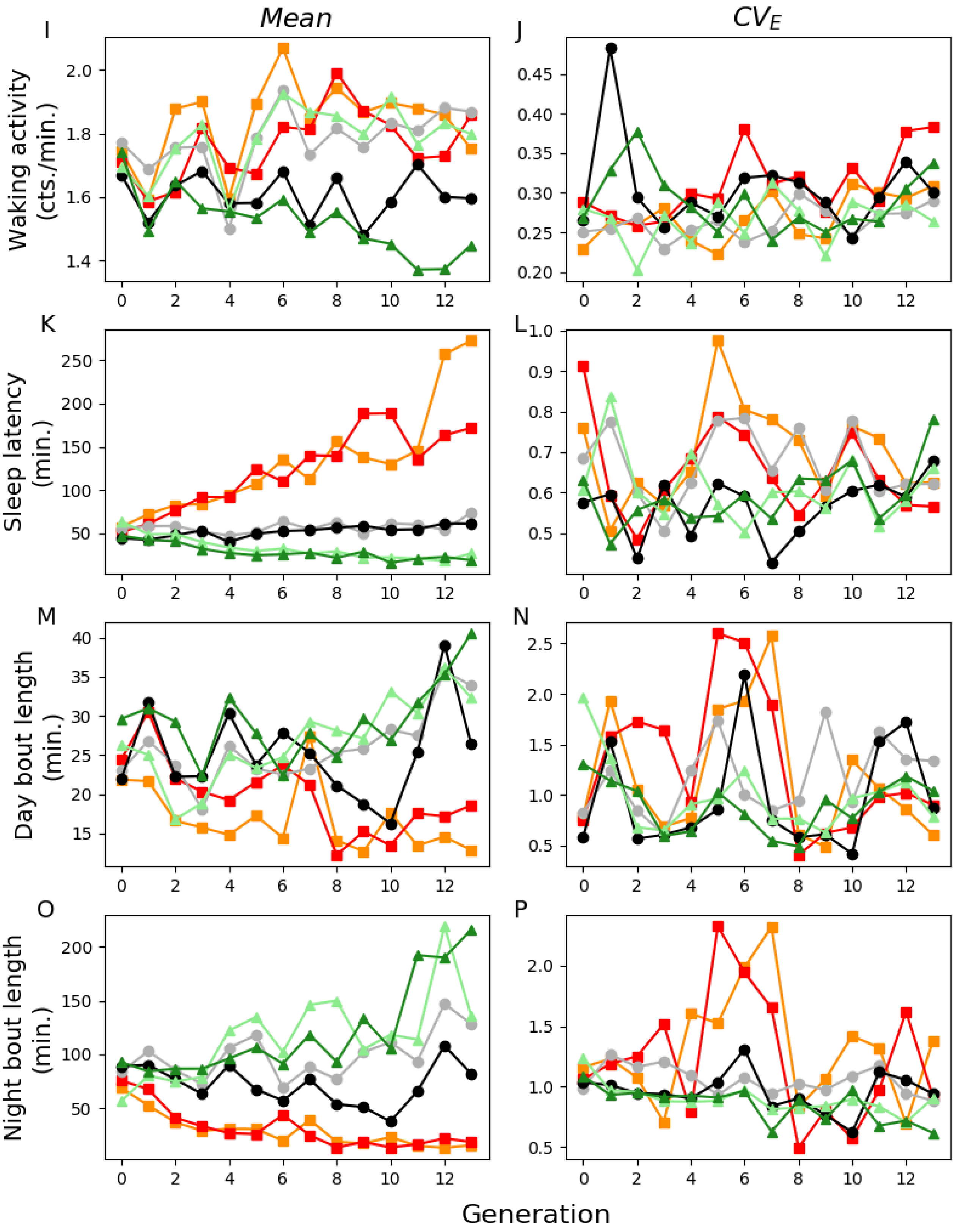

Mean
CVE
I
J
K
L
M
N
O
P
Waking activity (cts./min.)
Sleep latency (min.)
Day bout length (min.)
Night bout length (min.)
Generation

Q

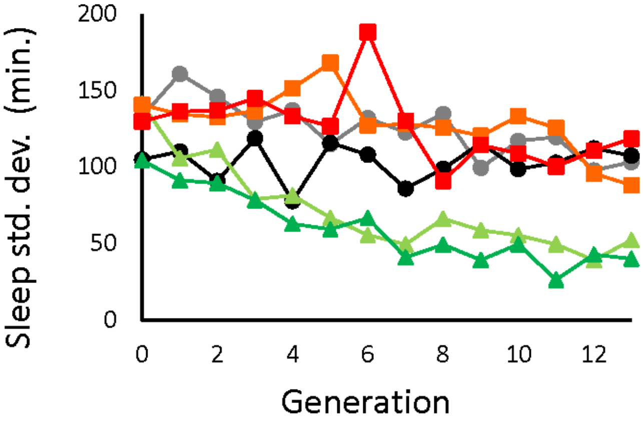

S2 Fig. Correlated response to selection for long/short night sleep and associated coefficient of environmental variation.

A, day bout number; B, day bout number coefficient of environmental variation CV_E;
C, day sleep; D, day sleep CV_E; E, night bout number; F, night bout number CV_E;
G, night sleep; H, night sleep CV_E; I, waking activity; J, waking activity CV_E; K, sleep latency;
L, sleep latency CV_E; M, day average bout length; N, day average bout length CV_E;
O, night average bout length; P, night average bout length CV_E; Q, night sleep standard deviation.
Light green, Replicate 1 long-sleeper population; Dark green, Replicate 2 long-sleeper population; O range, Replicate 1 short-sleeper population; Red, Replicate 2 short-sleeper population;
Gray, Replicate 1 control population; Black, Replicate 2 control population.
CV_E, coefficient of environmental variation.
