# Supplementary material for: Nonlinear expression patterns and multiple shifts in gene network interactions underlie robust phenotypic change in Drosophila melanogaster selected for night sleep duration
Source: PLoS Comput Biol. 2023 Aug 10;19(8):e1011389. doi: 10.1371/journal.pcbi.1011389 (PMC10443883; doi:10.1371/journal.pcbi.1011389)
Supplement: S3 Fig — A, long-sleeping Replicate 1; B, long-sleeping Replicate 2; C, short-sleeping Replicate 1; D, short-sleeping Replicate 2; E, control Replicate 1; F, control Replicate 2. (PDF) [file pcbi.1011389.s003.pdf]

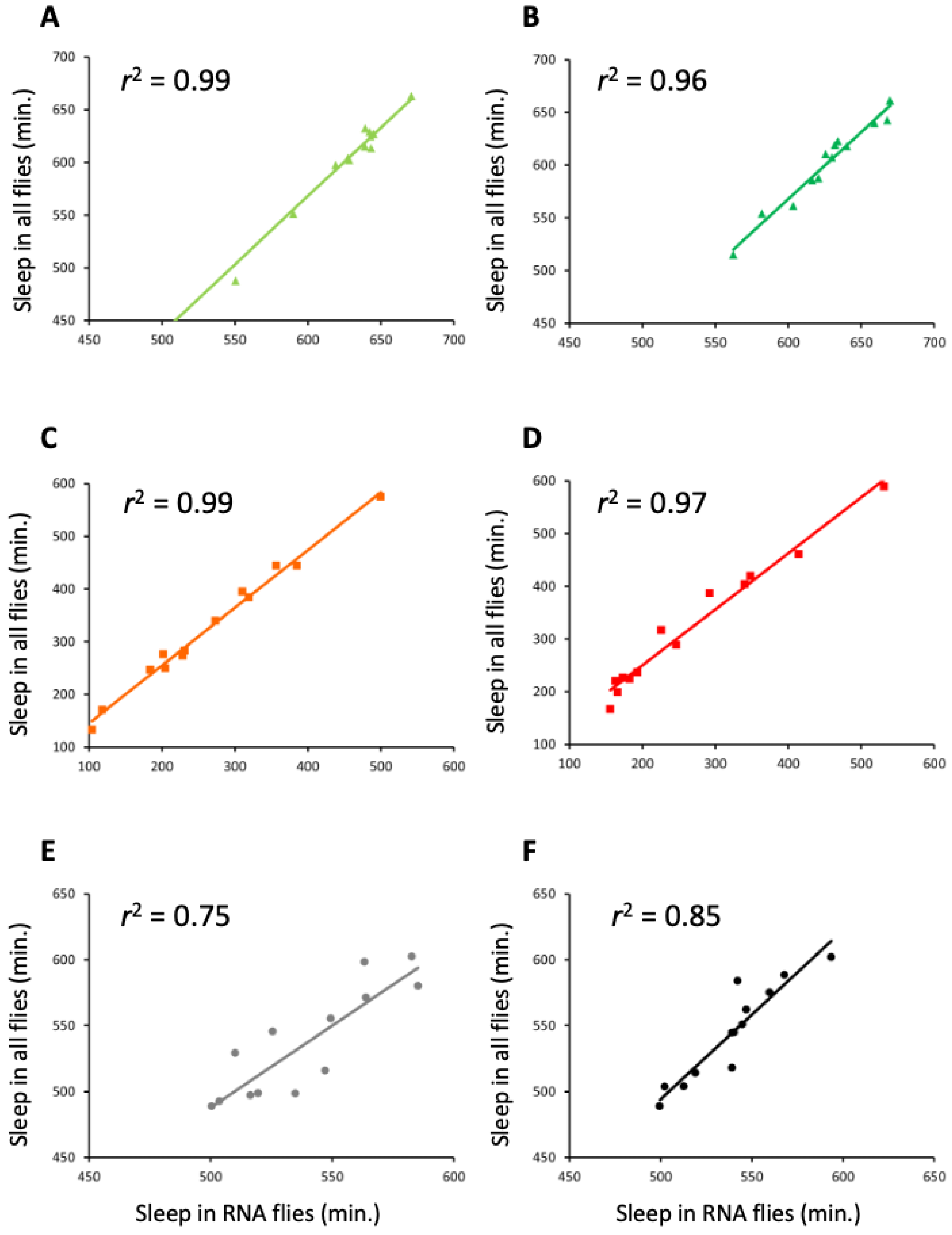

S3 Fig. Correlation of night sleep between flies harvested for RNA and all flies in the population. A, long-sleeping Replicate 1; B, long-sleeping Replicate 2; C, short-sleeping Replicate 1; D, short-sleeping Replicate 2; E, control Replicate 1; F, control Replicate 2.
