# Supplementary material for: Nonlinear expression patterns and multiple shifts in gene network interactions underlie robust phenotypic change in Drosophila melanogaster selected for night sleep duration
Source: PLoS Comput Biol. 2023 Aug 10;19(8):e1011389. doi: 10.1371/journal.pcbi.1011389 (PMC10443883; doi:10.1371/journal.pcbi.1011389)
Supplement: S4 Fig — A, fit of Gaussian Process model to pair of genes haf and CG1304; B, fit of Gaussian Process model to pair of genes CR43242 and CG1304; C, fit of single-channel Gaussian Process model to CG1304 gene; D, fit of single-channel Gaussian Process model to LysC gene. (PDF) [file pcbi.1011389.s004.pdf]

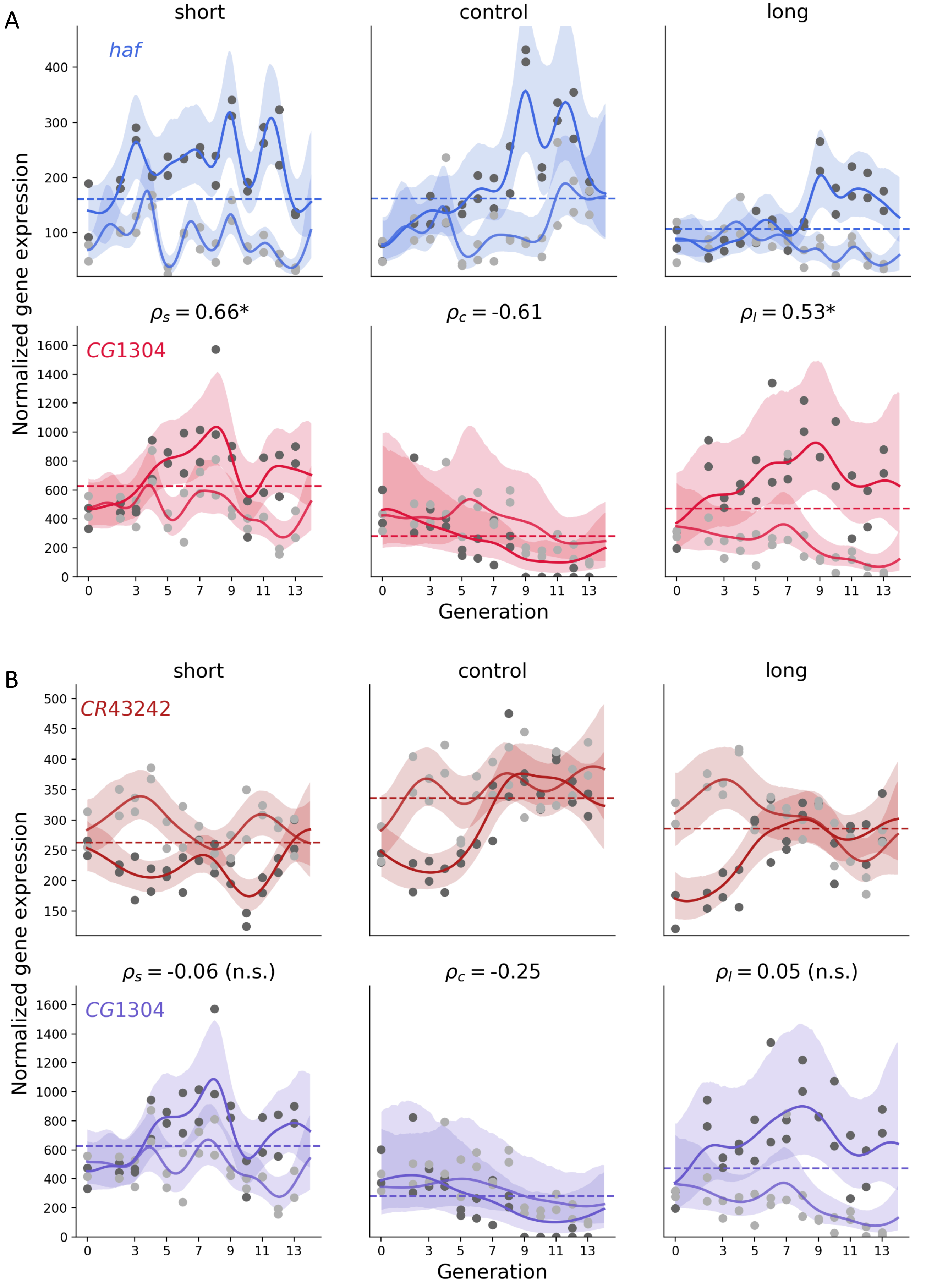
A
short
control
long
haf
Normalized gene expression
$\rho_s = 0.66$*
$\rho_c = -0.61$
$\rho_l = 0.53$*
CG1304
Generation
B
CR43242
$\rho_s = -0.06$ (n.s.)
$\rho_c = -0.25$
$\rho_l = 0.05$ (n.s.)
CG1304

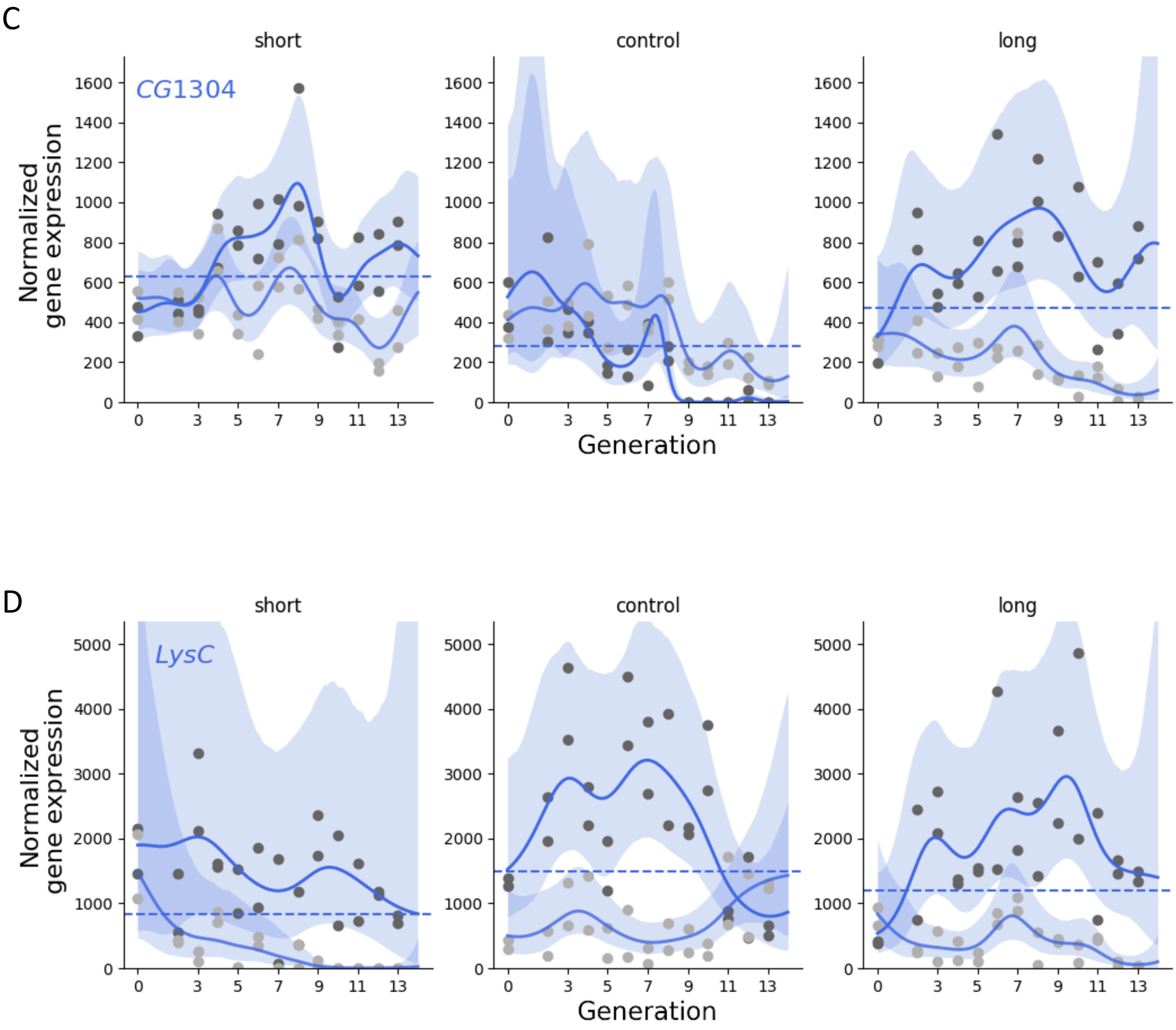

S4 Fig. Gaussian Process model fits to selected genes.

A, fit of Gaussian Process model to pair of genes *haf* and *CG1304*; B, fit of Gaussian Process model to pair of genes *CR43242* and *CG1304*; C, fit of single-channel Gaussian Process model to *CG1304* gene; D, fit of single-channel Gaussian Process model to *LysC* gene.
