# Supplementary material for: Nonlinear expression patterns and multiple shifts in gene network interactions underlie robust phenotypic change in Drosophila melanogaster selected for night sleep duration
Source: PLoS Comput Biol. 2023 Aug 10;19(8):e1011389. doi: 10.1371/journal.pcbi.1011389 (PMC10443883; doi:10.1371/journal.pcbi.1011389)
Supplement: S5 Fig — A, Males selected for short sleep; B, Females selected for long sleep; C, Females selected for short sleep. (PDF) [file pcbi.1011389.s005.pdf]

A

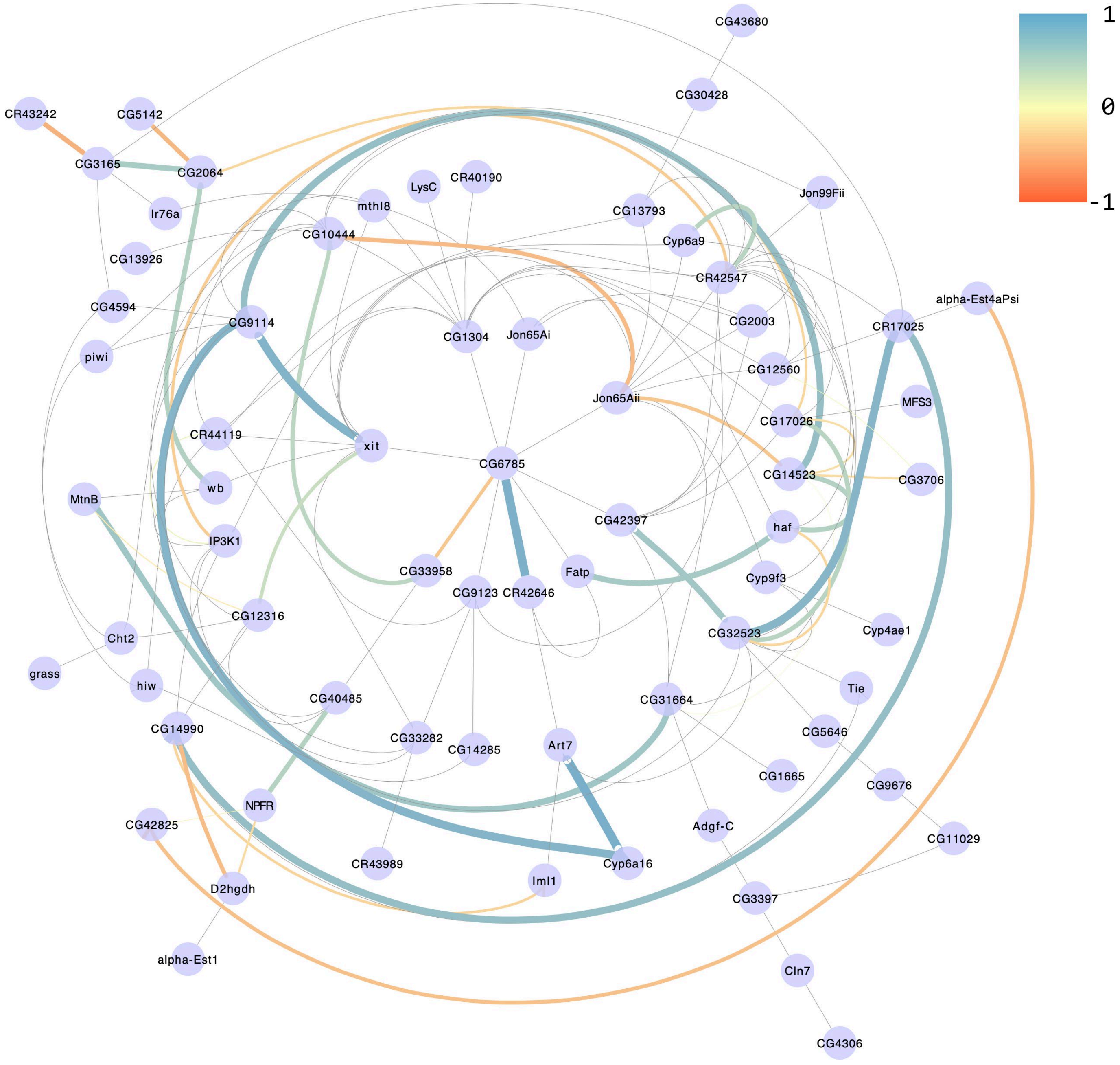
1
0
-1
CG43680
CG30428
CR43242
CG5142
CG3165
CG2064
Ir76a
LysC
CR40190
mthl8
Jon99Fii
CG13793
CG10444
Cyp6a9
CG13926
CR42547
CG4594
alpha-Est4aPsi
CG9114
CG1304
Jon65Ai
CG2003
CR17025
piwi
CG12560
Jon65Aii
MFS3
CR44119
CG17026
xit
CG6785
CG14523
CG3706
wb
MtnB
CG42397
haf
IP3K1
Fatp
CG33958
Cyp9f3
CG9123
CR42646
CG12316
Cyp4ae1
CG32523
Cht2
grass
hiw
Tie
CG40485
CG31664
CG14990
CG33282
CG5646
Art7
CG14285
CG1665
CG9676
NPFR
CG42825
Adgf-C
CG11029
CR43989
Cyp6a16
Iml1
D2hgdh
CG3397
alpha-Est1
Cln7
CG4306

B

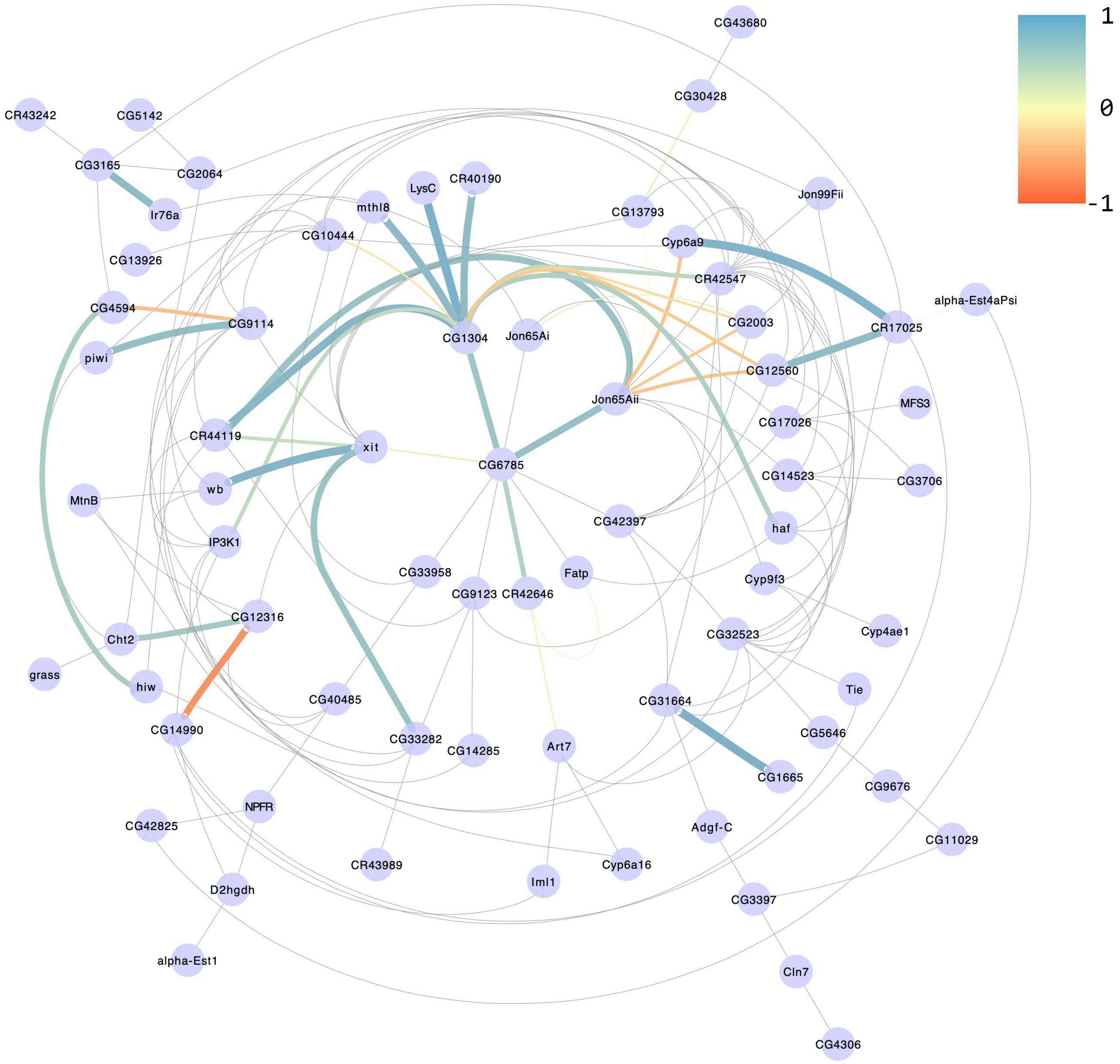

1
0
-1
CG43680
CG30428
CR43242
CG5142
CG3165
CG2064
Ir76a
LysC
CR40190
mthl8
CG13793
Jon99Fii
CG10444
Cyp6a9
CG13926
CR42547
alpha-Est4aPsi
CG4594
CG9114
CG2003
CR17025
piwi
CG1304
Jon65Ai
CG12560
Jon65Aii
MFS3
CG17026
CR44119
xit
CG6785
CG14523
CG3706
wb
MtnB
CG42397
haf
IP3K1
Fatp
CG33958
Cyp9f3
CG9123
CR42646
CG12316
Cyp4ae1
CG32523
Cht2
grass
hiw
CG40485
CG31664
Tie
CG14990
CG33282
CG14285
Art7
CG5646
CG1665
CG9676
NPFR
CG42825
Adgf-C
CG11029
CR43989
Cyp6a16
Iml1
D2hgdh
CG3397
alpha-Est1
Cln7
CG4306

C

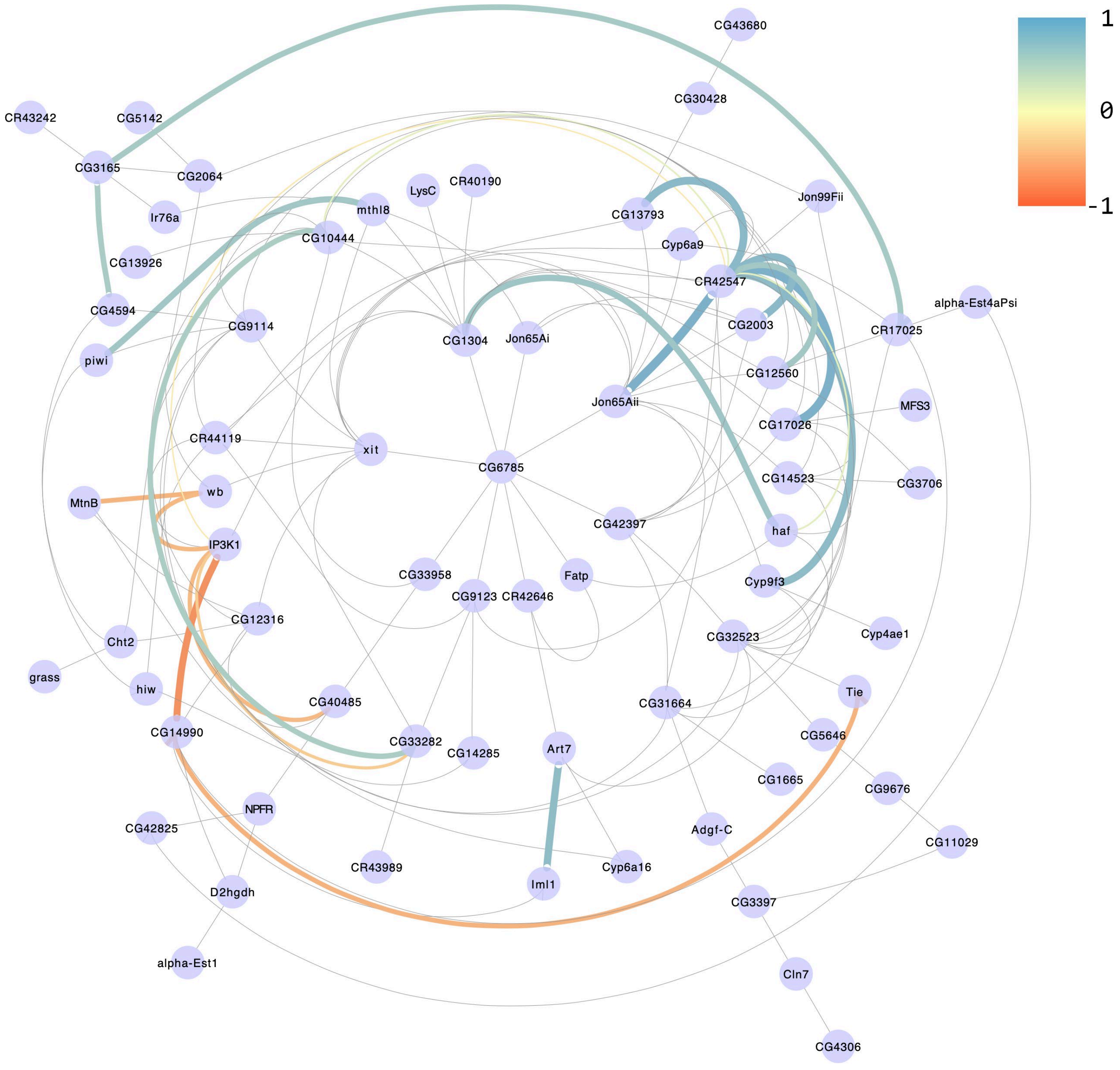

S5 Fig. Gene interaction networks.

A, Males selected for short sleep; B, Females selected for long sleep; C, Females selected for short sleep.
