# Supplementary material for: Nonlinear expression patterns and multiple shifts in gene network interactions underlie robust phenotypic change in Drosophila melanogaster selected for night sleep duration
Source: PLoS Comput Biol. 2023 Aug 10;19(8):e1011389. doi: 10.1371/journal.pcbi.1011389 (PMC10443883; doi:10.1371/journal.pcbi.1011389)
Supplement: S6 Fig — For each candidate gene, the gene expression in the Minos mutant and corresponding control are plotted. * or #P < 0.05 by Kruskal-Wallis test. A, CG12560; B, Jon65Aii; C, CG13793; D, Cyp6a16; E, hiw. (PDF) [file pcbi.1011389.s006.pdf]

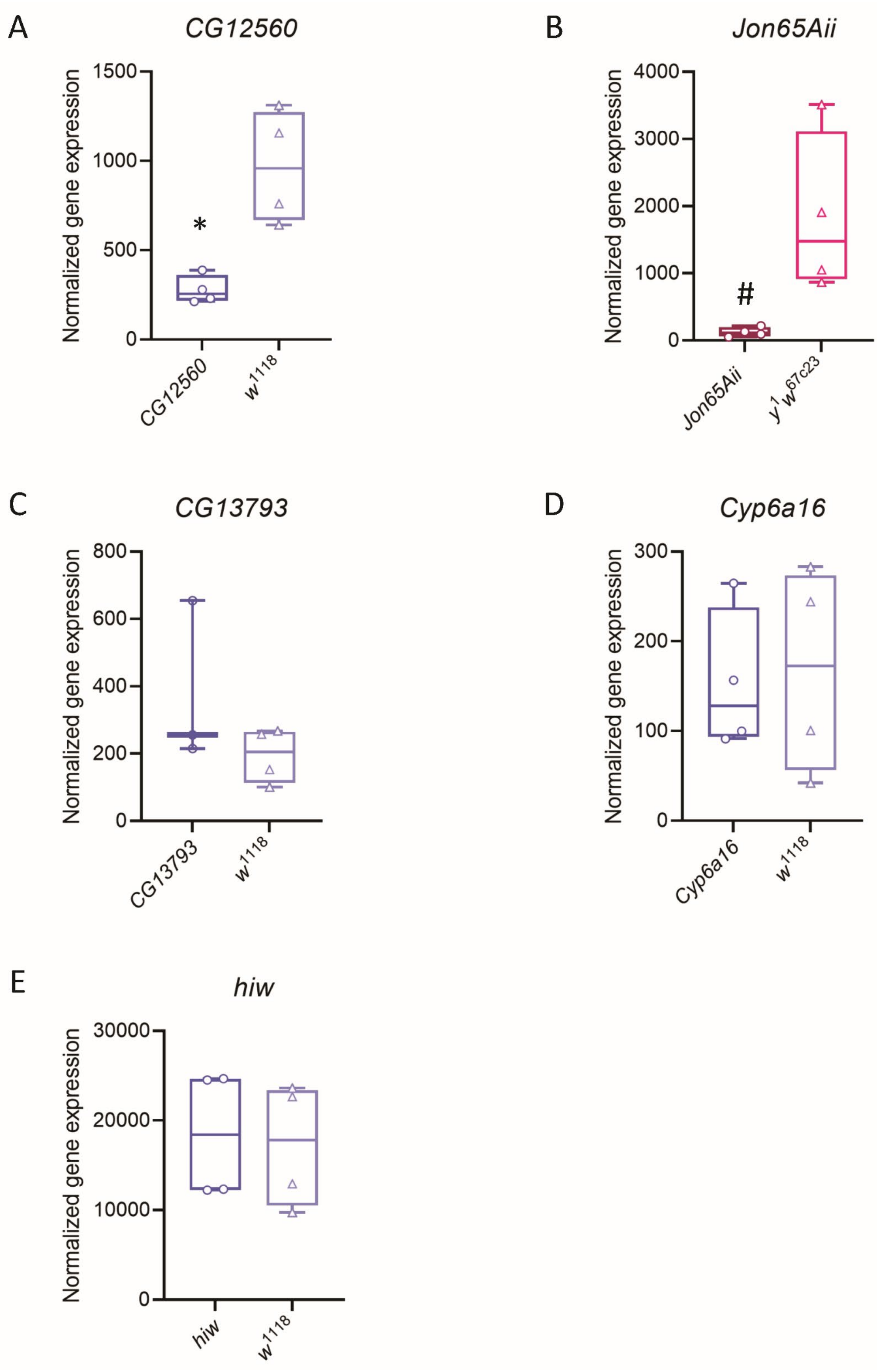

S6 Fig. Gene expression in *Minos* mutants.

For each candidate gene, the gene expression in the *Minos* mutant and corresponding control are plotted. * or #, *P* <0.05 by Kruskal-Wallis test. A, *CG12560*; B, *Jon65Aii*; C, *CG13793*; D, *Cyp6a16*; E, *hiw*.
