# Supplementary material for: Nonlinear expression patterns and multiple shifts in gene network interactions underlie robust phenotypic change in Drosophila melanogaster selected for night sleep duration
Source: PLoS Comput Biol. 2023 Aug 10;19(8):e1011389. doi: 10.1371/journal.pcbi.1011389 (PMC10443883; doi:10.1371/journal.pcbi.1011389)
Supplement: S7 Fig — A, CG13793 females; B, CG13793 males; C, Cyp6a16 males; D, hiw females; E, hiw males. (PDF) [file pcbi.1011389.s007.pdf]

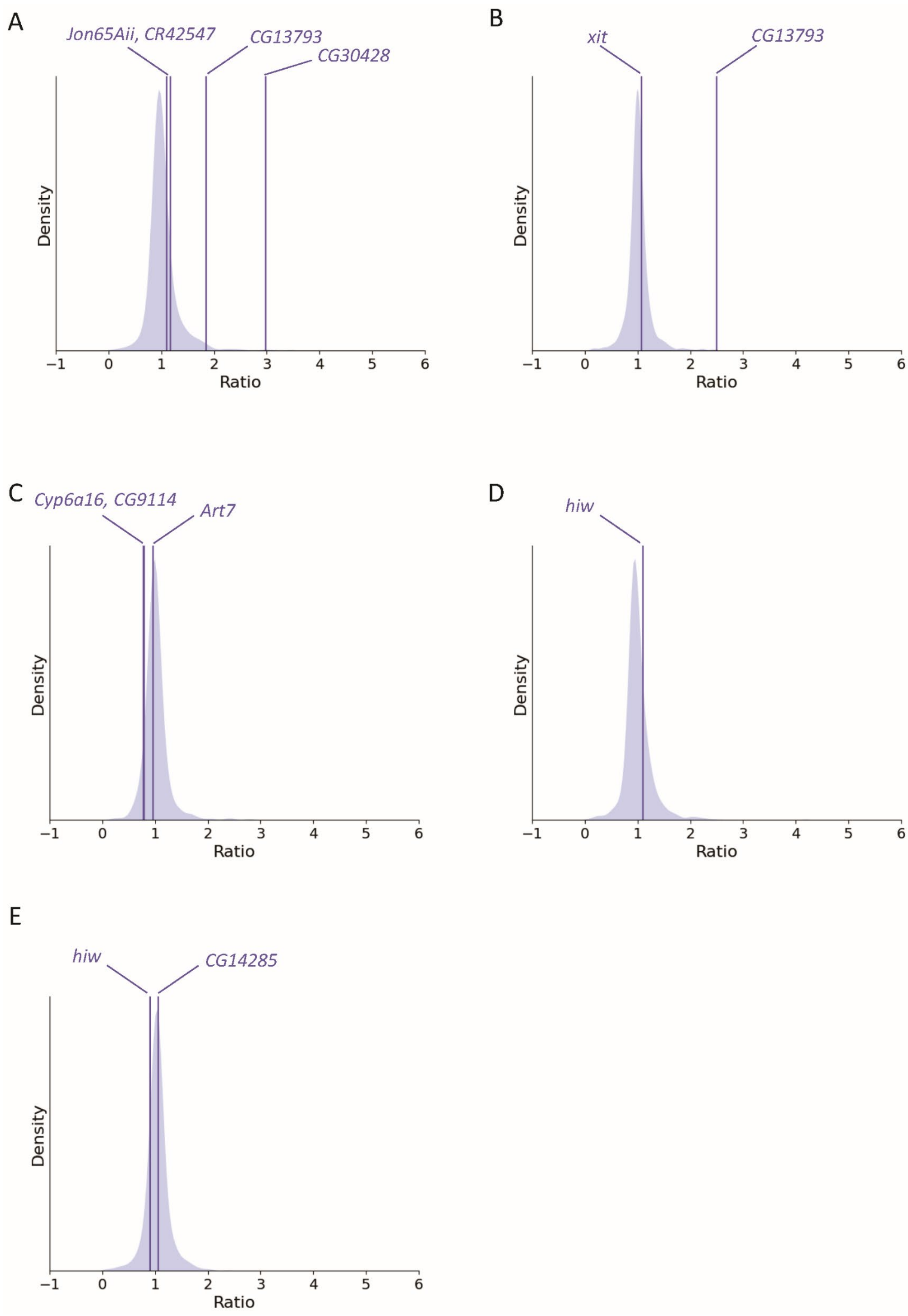

S7 Fig. Comparison of ratios of gene expression between genes with significant Gaussian Process correlations and unrelated genes for *CG13793*, *Cyp6a16*, and *hiw Minos* mutants.
A, *CG13793* females; B, *CG13793* males; C, *Cyp6a16* males; D, *hiw* females; E, *hiw* males.
