# Supplementary material for: Nonlinear expression patterns and multiple shifts in gene network interactions underlie robust phenotypic change in Drosophila melanogaster selected for night sleep duration
Source: PLoS Comput Biol. 2023 Aug 10;19(8):e1011389. doi: 10.1371/journal.pcbi.1011389 (PMC10443883; doi:10.1371/journal.pcbi.1011389)
Supplement: Attachment — Submitted filename: Review_Souto-Maior_et_al_v1.pdf [file pcbi.1011389.s025.pdf]

Summary

In the manuscript entitled "Nonlinear expression patterns and multiple shifts in gene network interactions underlie robust phenotypic change in Drosophila melanogaster selected for night sleep duration", the authors used a Gaussian Process (GP) model to systematically identify changes in interactions between genes over the generations of artificial selection. Specifically, they conducted an artificial selection experiment with Drosophila melanogaster, and, every generation, they harvested RNA from files. Then, they established a multi-channel GP model for estimation of the nonlinear expression patterns of genes related to sleep phenotypic change, which is impossible with a linear model analysis as mentioned in the manuscript. Using the GP model, they identified some genes whose expression level significantly changes in the artificial selection. Lastly, to validate part of the findings obtained using the GP model, mutational analyses were performed by a *Minos* insertion. These results clearly demonstrate the usefulness of the GP-based approach in investigating the complex molecular interactions leading to diverse phenotypes. I believe that the approach is interesting and correctly applied. Considering this, the current manuscript well fits the scope of the *PLOS Computational Biology*, and thus I recommend the publication. However, I provide major and minor comments to be solved before the publication in below.

Major comments

1. To understand this work, readers must have both experimental and mathematical/statistical backgrounds, as Reviewer 1 mentioned. However, several potential readers of this work, such as evolutionary biologists and clinician in sleep research field might not have enough mathematical/statistical backgrounds. I believe that this problem frequently occurs in interdisciplinary research. I felt that, to circumvent this problem, the authors described their methodology in detail in the Methods and Materials, which is good. However, even with the detailed description, the manuscript can lose potential readers due to the inherent difficulties of the methodology, making readers a little bit stressful. To completely avoid this circumstance, I kindly recommend that the authors provide a user-friendly computer package to implement their GP algorithm. Here, user-friendly means that users do not need to understand all the code, and, by just changing the input (e.g., RNA-seq data), they can run the code and analyze their own data. After this, it would be great if the authors clearly mention that they provide an easy-to-use and user-friendly computer package for implementation of the algorithm, for example, in the introduction section.

2. Approaches based on GP models have been recently used to infer dynamics of complex biological systems. Specifically, Yang et al. (Yang et al. PNAS 2021 PMID: 33837150) proposed a method called MAGI that use a Gaussian process model over time series data. It would be great if the authors can clearly describe advantages (or novelty) of the method proposed in the manuscript compared to the MAGI in the manuscript.

3. Because the authors said "*we develop a flexible Gaussian Process model* …" in the abstract, and the manuscript focuses on the application of the method to experimental data, I believe that the authors need to describe previous approaches of network analysis potentially related to the proposed GP method (e.g., Langfelder & Horvath BMC Bioinformatics 2008 PMID: 19114008; Yang et al. PNAS 2021 PMID: 33837150; Horvath & Dong PLOS Comp, Biol. 2008 PMID: 18704157) in the introduction section. Moreover, it would be great if the authors can more clearly describe the novelty of this work. Specifically, if there is a methodological advance in the proposed method compared to the previous ones, please clearly describe it. If the main contribution of the manuscript is a specific application of the previously

developed GP method and identification of previously unknown biological knowledge, please more focus on describing the biological findings instead of describing the adopted methodology.

Minor comments

1. As the author described in the Methods and Materials, 10 files were chosen from 75 files and frozen for RNA extraction at ZT6. Among the extracted RNAs, levels of some RNA might oscillate over time, as the Review 2 mentioned. Thus, the conclusion of the manuscript may change depending on the collection time of RNA. It would be great if the authors discuss this point in the discussion section in addition to mentioning the collection time in the Methods and Materials. At least, it might be needed to mention that the future work that checks whether the findings are preserved even with the samples collected at different times in the manuscript.

2. I could not access the Git Hub repository via the link provided in the Data Availability section. It would be great if the authors can check whether the link works.

3. I believe that this technique can be applied to other biological systems, such as gene regulatory networks associated with Fatty acid Composition (Basnet et al. Plant Physiol. 2016 PMID: 26518343) and cell signaling networks associated with antibiotic stress (Kim et al. Science Advances 2022 PMID: 35302852). It would be great if the authors mention this point in the discussion section.
