# Supplementary material for: Nonlinear expression patterns and multiple shifts in gene network interactions underlie robust phenotypic change in Drosophila melanogaster selected for night sleep duration
Source: PLoS Comput Biol. 2023 Aug 10;19(8):e1011389. doi: 10.1371/journal.pcbi.1011389 (PMC10443883; doi:10.1371/journal.pcbi.1011389)
Supplement: Attachment — Submitted filename: 131933_1_rebuttal_2579033_rp5f47.docx [file pcbi.1011389.s024.docx]

**Manuscript number:** RC-2022-01758

**Corresponding author(s):** Harbison, Susan and Souto-Maior, Caetano

*[Please use this template only if the submitted manuscript should be considered by the affiliate journal as a full revision in response to the points raised by the reviewers.*

*If you wish to submit a preliminary revision with a revision plan, please use our "*[*Revision Plan*](https://docs.google.com/document/u/0/d/1HHp62Tfeb44nd3Gu_3ChB0b8gmMdEog7GjZcZFwHSiQ/edit)*" template.* ***It is important to use the appropriate template to clearly inform the editors of your intentions.****]*

## 1. General Statements [optional]

*This section is optional. Insert here any general statements you wish to make about the goal of the study or about the reviews.*

We thank the reviewers for their time and care in evaluating our manuscript. They raise several important points, which we have addressed, resulting in a greatly improved manuscript. Please note that we numbered the comments from both reviewers for ease of reference, as we cross-referenced comments in some cases. Reviewer comments are in italics; our responses are provided in blue plain text.

## 2. Point-by-point description of the revisions

*This section is mandatory.* *Please insert a point-by-point reply describing the revisions that were already carried out and included in the transferred manuscript.*

Reviewer #1 (Evidence, reproducibility and clarity (Required)):

*Summary*:

*The authors of this work generated a Sleep Advanced Intercross Population from 10 extreme sleeper Drosophila Genetics Reference Panel. This new out-bred population was subjected to a artificial selection with the aim of understanding the genes underlying the sleep duration differences between three populations: short-sleep, unselected, and long-sleep. Using analysis of variance the authors identified up to nearly 400 of genes that were significant selected over the various generations and showed opposite trends for long and short sleep, thus potentially relevant for the regulation of sleep duration. 85 of these genes were consistent between male and females sub-populations, suggesting a small number of genetic divergences may underlie sex-independent mechanisms of sleep.

Given the time-course nature of the generational data obtained, the authors studied potential correlations and interactions between these 85 identified candidate genes. Initially, the authors used pairwise Spearman correlation, noticing how this method could not filter most of pairwise interaction (around 40% of all possibilities were significant). To overcome the linear limitations of the previous approach, the authors implemented a more complex, non-linear Gaussian process model able to account for pairwise interactions. This new approach was able to identify a smaller number of different, and potentially more informative, correlations between the candidate genes previously identified.

Lastly, with genetic manipulations, the authors show in vivo that a subset of the candidate genes is causally related with the sleep duration as well as partially validating some of the correlation identified by their new model.

The authors conclude that, given the non-linear and complex nature of biological systems, simplistic linear approaches may not suffice to fully capture underlying mechanisms of complex traits such as sleep.

*Major comments**

1. *Most of the the work presented focus on the computational and statistical analysis of different populations submitted (or not) to a process of artificial selection for short or long sleep duration. As such, the amount of potentially relevant biological conclusions to be tested is mostly unfeasible. The authors already present additional experiments to partially support some, though not all, of their findings. Given the manuscript is written as a method innovation, these additional experiments illustrate the potential uses of the method described.*

**Our response:** The reviewer raises a very important point, one that is at the very impetus of our work. We agree that it is not possible to test all combinations of genes in all contexts to determine whether they influence sleep or not. In contrast to the situation for circadian rhythms, where the core clock is controlled by just four genes, recent work has concluded that sleep is a set of complex traits influenced by large numbers of genes. Robust computational methods are needed to identify the complex interactions among genes. The current manuscript is a first step towards achieving this goal.

*(OPTIONAL) However, since the one of the focuses of this work in identifying potential gene interactions, it would be interesting if the authors could test a "double knockout" and perhaps demonstrate evidence for epistasis between two of the identified genes. Having access to single mutants, this experiment should be realistic. However, I have no hands-on experience working with Drosophila and I am unable to accurately estimate the amount of resources and time such and experiment could take. My initial guess would be 3-6 months work should suffice.*

**Our response:** The reviewer makes an interesting proposal. While such an experiment would provide some additional information, our method does not make any prediction about what a double knockout would do, either to the sleep phenotypes or to gene expression.

2*. In regards to the gene CG1304, it seems to be an important example used throughout the manuscript. It should be carefully re-analyzed as was considered for interaction analyses without showing opposite trends for short- and long-sleep populations (see minor comments on figure 2).*

**Our response**: We are not entirely certain that we understand the reviewer’s point. We note that significant genotype-by-selection-scheme interactions may not manifest as opposite trends and this is not what is being tested for significance. The likelihood ratio is a test for a significant effect of including sel x gen coefficients for **both** short and long schemes; therefore, GLM significance may mean that either one or the two selection schemes are significantly different from controls, not from each other. We could, for instance, apply three different tests: one **(i)** comparing between long and short flies; the second **(ii)** comparing short flies to controls; and the third **(iii)** comparing long to controls and find that the first test is significant — i.e. short is different from long — and that the two others are not — i.e. neither scheme is found to be different from controls. The opposite could also happen: short and long flies may not be different from each other, but with both being different from controls.

Since we are interested in identifying differences of either to controls, our choice of statistical test is equivalent to performing tests **(ii)** and **(iii)** without the need to perform and correct for multiple tests. While there are caveats to this choice (like all choices), linear model-based differential expression analysis has its own caveats, and has limited ability to pick up arbitrary trends, so it serves as a coarse-grained filter for large shifts since it’s too costly (computationally) to run the Gaussian process on 50 million pairwise combinations.

3. *One major comment would be that the claim that the Gaussian process method is more sensitive and specific than simpler approaches, though intuitively understandable, does not seem to be fully correct from a strict statistical point of view, given the lack of a gold standard reference to compare if the new method is indeed picking more true positives/negatives. I would reconsider re-rephrasing such statement in the absence of a biologically relevant validation set.*

**Our response:** We agree with the reviewer that there is no ‘gold standard’ reference data set with which to compare our findings. We have softened this language a bit in response, where it occurs in both the Abstract and the Results.

Under Abstract, we changed “Our method not only is considerably more specific than standard correlation metrics but also more sensitive, finding correlations not significant by other methods” to “Our method appears to be not only more specific than standard correlation metrics but also more sensitive, finding correlations not significant by other methods.”

Under Results, we changed “Therefore, computing correlations between genes using covariance estimates from the Gaussian Processes greatly increases specificity over direct correlations. Furthermore, the Gaussian processes are not only more specific but more sensitive…” to “Therefore, computing correlations between genes using covariance estimates from the Gaussian Processes appears to increase specificity over direct correlations. Furthermore, the Gaussian Processes appear to be more sensitive…”

4. *Finally, the study appears to be well powered and it is clear that the authors were careful in their explanation of the statistical methods. However, I could not find the copy of the code/script used for the model. Without it, it would be very difficult to fully reproduce the results as both the language used (Stan) and the method itself are not common in the sleep research field.*

**Our response:** We thank the reviewer for noticing this, and apologize for this oversight. The code used for analysis has been deposited in GitHub under: https://github.com/caesoma/Multiple-shifts-in-gene-network-interactions-shape-phenotypes-of-Drosophila-melanogaster.

We have noted the script location in the Data Availability statement. We added a statement to read “All scripts used for the model have been deposited in Git Hub https://github.com/caesoma/Multiple-shifts-in-gene-network-interactions-shape-phenotypes-of-Drosophila-melanogaster.”

**Minor comments**

5. *The statistical cut-off used for gene expression hierarchical GLMM after BH correction was of 0.001, which is 50 times more strict than the common 0.05. Could the authors comment on how this choice may impact the results compared to those available in the literature and on the rational for choosing such a value.*

**Our response:** A FDR of 0.05 would increase the number of genes identified (3,544 for females; 1,136 for males, with 462 overlapping). The FDR of 0.001 is consistent with the lowest threshold typically used for gene expression data collected during other artificial selection experiments (Mackay et al., 2005; Morozova et al., 2007; Edwards et al., 2006), though thresholds as high as 0.20 have been used (Sorensen et al., 2007). We have added to the last statement to the Methods and Materials section under “Generalized Linear Model analysis of expression data” to read “Model p-values were corrected for multiple testing using the Benjamini-Hochberg method (Benjamini and Hochberg, 1995), with significance defined at the 0.001 level, consistent with the lower threshold applied in other artificial selection studies (Mackay et al., 2005; Morozova et al., 2007; Edwards et al., 2006).”

6. *Heritability calculations are not mentioned in the methods. Could it be useful to include a small paragraph? Could a small comment be done on the differences in h2 for the short sleep replicates which show ~10x difference?*

**Our response:** We thank the reviewer for noticing this omission and apologize for the oversight. We have added the following statements to the Methods and Materials under “Quantitative genetic analyses of selected and correlated phenotypic responses.”

“We estimated realized heritability h^2^ using the breeder’s equation:

$$h^{2}= \frac{\sum R}{\sum S}$$

where $\sum R$ and $\sum S$ are the cumulative selection response and differential, respectively (Falconer and Mackay, 1996). The selection response is computed as the difference between the offspring mean night sleep and the mean night sleep of the parental generation. The selection differential is the difference between the mean night sleep of the selected parents and the mean night sleep of the parental generation.”

Additionally, we thank the reviewer for noticing the large difference in the realized heritability between the short sleeping population replicates; the heritability for replicate 1 is a typo and should be 0.169, not 0.0169. Hence, the heritabilities of both replicate populations are quite similar, i.e., 0.169 for replicate 1 and 0.183 for replicate 2. We have corrected this error in the Results.

7. *In regards to the model implementation, what would be the implications of not enforcing positive semi-definiteness on the co-variance matrix, given than that these are strictly positive semi-defined?*

**Our response:** All covariance matrices are by definition positive semi-definite (PSD), since they cannot yield negative values for the probabilities associated to them, so it would not be possible to relax that assumption generally. The only choice we could make would be on the number of genes included (M) in each multi-channel gaussian process model, and this in turn would by design enforce positive semi-definiteness on an matrix of size MN, (N being the number of generations). As noted in the appendix, “enforcing” positive semi-definiteness on smaller blocks of a larger 2D-array of covariances (which is not itself a covariance matrix) does not imply the latter is PSD and therefore seems like a softer constraint. In practice scaling up to a model where M >> 40 is not trivial from a computational and inference point of view, so the choice of smaller M is in a way imposed on us, and fortunately it is the less limiting one. We provide the appendix as a general clarification on the subtleties of Gaussian Processes, but a comprehensive assessment is beyond the multidisciplinary scope of this article and would require a narrower mathematical/statistical description in a standalone methodological article or technical note.

8. *The methods mention that PCA projection were performed on the first 3 components, however only the first two are showed.*

**Our response:** PCA was performed on 10 components, although the algorithms will commonly compute all components and return only the selected number. The variance of the third component is smaller than ~5% (that of the second PC). In practice PC1 is by itself enough to show the clear separation of expression per sex with ~65% of the variance; PC2 is in fact only shown to improve visualization. Plots of the remaining components will not show clear separation among samples as the variance explained is so small. We have corrected the Methods to indicate that PCA was performed on 10 components rather than 3.

9. *Figure 1 refers to the mean night sleep time of the population. Could some measurement of variability (se or sd) be represented to provide a general idea of the distribution of the values? Additionally, the standard deviation of associated with the CVe estimates are mentioned but not showed explicitly. Could they maybe be added to the text as to illustrate how much such deviations were reduced?*

**Our response:** We thank the reviewer for this comment. Including either the standard errors or standard deviations on the plot of the response to selection (Figure 1A) makes visualization unwieldy; thus we have added an additional supplemental table, Supplementary Table S15, that contains the mean night sleep, standard deviation, and number of flies measured for each generation in each replicate population. We also added a plot of the standard deviation in night sleep per generation to Supplemental Figure S2 (letter “Q” in the figure) so that the reduction over time in each population can be seen.

Under “Data Availability,” We added the following: “Night sleep phenotypes per selection scheme/sex/generation/population replicate are listed in Table S15.”

10. *Figure 2 shows the linear model fits for gene CG1304. I find this gene on the list of significant genes for both sexes (tables S5/6), but it does not seem to be one that shows opposite trend for short- and long-sleep (tables S7/8). Surprisingly, it shows up again on table S10! However, the text introducing the figure reads like this should be one of the 85 sex-independent genes. Would it be best to provide an example of what a significant gene looks like?*

**Our response:** As mentioned in our response to comment #2 above, significance in the likelihood-ratio test does not imply opposite trends between long and short selection schemes, but between a model that includes specific slope coefficients for selection scheme by generation (both long and short) compared to a reduced model where the only slope is one associated to generation and therefore independent of selection scheme.

11*. Figure 3 would be interesting to have both the GP correlations and the Spearman correlations to illustrate the methodological differences. I would be curious to see at least one pairwise expression scatter-plot as well just to see how they correlate in one plot.*

**Our response:** Table S11 contains all (significant and nonsignificant) GP and Spearman values side-by-side for comparison. High correlations are likely to conform to the Spearman assumptions of a monotonic relationship; nevertheless, this will not be so for the majority of genes since the difference in the number of Spearman and GP-significant genes is tenfold or more, so it would be misleading to focus on individual-gene relationships without taking into consideration the transcriptome wide results for any method employed.

We would like to stress that there is nothing particularly special about CG1304 in and of itself; furthermore, there are no “representative” genes or figures in this manuscript. Instead, CG1304 is chosen because its GLM and GP fits are illustrative of the limitations and capabilities of each model to pick up certain kinds of trends, and especially because it is especially instructive of how correlations arise from the GP model, which may not be intuitively clear to all readers*.*

12*. Figures 3S3/4 are described as showing single- and multi-channel models don't change substantially. Would this be expected and why?*

**Our response:** This is not necessarily expected, as scaling up from a single to a multi-channel model will add additional parameters as well as constraints, like positive the semi-definiteness mentioned in comment #7 above. If that seemed to have considerable impact on the fits it could challenge our assumption that the signal variance parameters estimated from the single-channel are good priors for the same parameters in the two-channel model (although this is not a hard constraint, so in the worst case the result could still only be a slight bias).

13. *Having build different networks of pairwise associations of genes (projecting on a unified network as illustrated on figure 5), it could in interesting to compare the network topologies at a basic level such as node degrees, overlapping sub-networks, are they potentially scale free as previously described for biological systems, etc.*

**Our response:** The reviewer makes an interesting point. Indeed summaries of the network could be useful information about the system level parameters, which are the main results of this paper. We now include the number of connections (i.e., the degree) to each gene in each of the four networks presented in Figure 5 in a new supplemental Table (Table S13). We also plot the distribution of node connectivity below. The distributions do not appear random (i.e., a normal distribution), and appear closer to a power-law or scale-free distribution. However, the small size and low average degree of these networks make a formal test unfeasible, and a recent study suggests that a log-normal distribution is in general more likely than a power-law distribution (Broido et al., Nat Comm, 2019), so we lack the evidence to claim that these networks are scale-free.

We have added to the Results under “Gaussian Process model analysis uncovers nonlinear trends and specifically identifies covariance in expression between genes”: “Table S13 lists the number of connections (degrees) that each gene has with others in the network. The average number of connections for long-sleeper males was 2.6; the other three networks had average degrees of 2.0 or less (2.0 for long-sleeper females and short-sleeper males; 1.75 for short-sleeper females).”

14. *On table S6 I noticed some gene symbols were loaded as dates (1-Dec)*

**Our response:** We thank the reviewer for noticing this, the gene symbol is supposed to be dec. We have corrected this in Table S6 (now Table S7).

15. *In results, the phenotypical response to artificial selection is sometimes described in minutes, other times in hours. Though this is an hurdle, it could make the values easier to compere if they were consistently formatted as minutes (hours).*

**Our response:** We are unsure what the reviewer is referring to. We only see one sentence in which we used hours, and that was the concluding sentence under Results, “Phenotypic response to artificial selection.” The remainder of the manuscript refers to sleep times in minutes, phenotypes in all of the figures are plotted as minutes, and all of the supplemental material refers to times in minutes.

16*. Over 99% of chains converged after three runs. Even though the reasons for the lack of convergence of these chains was not investigated, could this be a relevant effect? 1% of 3570 interactions is still 35 potential interactions. Do the non convergent chains relate with specific genes?*

**Our response:** Bayesian MCMC inference is a stochastic algorithm, so there is a finite chance that any given run doesn’t converge, and that means that all eight parallel chains must converge and mix as measured by the stringent choice of R-hat metric being within 0.05 of unity. Relaxing the interval to 0.1 or 0.2 could still be acceptable, but we made the choice of a stringent threshold to avoid making interpretations on less-than-ideal runs. There is no evidence that there is any gene-specific problem, usually it would be one out of eight chains that would not mix well and throw off the diagnostic metrics (like relaxing the metrics, an acceptable approach could be accepting a run with 6-7 chains converging properly, but we decided to rerun all chains and only accept 100% convergence but accept a possible loss). Non-converging/nonmixing runs are likely to eventually do so, but since were are running tens of thousands of runs (3570 pairwise combinations × 3 schemes × 8 chains) a massively parallel implementation in a HPC cluster is required. Finally, seeing that 145 is ~4% of the total number of interactions, a naïve expectation would be that no more than one interaction would come out significant — while there is a chance that an interesting interaction was identified, the same can be said for potential false negatives computed using the GLM, which is a consequence of working at a high-throughput scale.

17. *The GO terms identified as significantly enriched after pvalue correction point to a clear association of the 85 genes identified with Serine proteases. Could this be discussed further to highlight biological findings of the work in the context of neuronal function or sleep regulation?*

**Our response:** The reviewer is correct, nine putative Serine proteases are significantly enriched among the 85 genes. All nine exhibit some expression in neurons and in epithelial cells, and all are expressed at the adult stage. The appearance of these enzymes is interesting given their role in proteolysis.

We have updated the Discussion to read, “Interestingly, our Gene Ontology analysis identified nine genes from the 85-gene network with predicted Serine endopeptidase/peptidase/hydrolase activity: CG1304, CG10472, CG14990, CG32523, CG9676, grass, Jon65Ai, Jon65Aii, and Jon99Fii. All of these genes are expressed in neurons and epithelial cells, and all genes are expressed at the adult stage (Li et al., 2022). Serine proteases are a large group of proteins (257 in Drosophila) that perform a variety of functions (Cao and Jiang, 2018). Their predicted enzymatic activity suggests a putative role in proteolysis. This is an intriguing observation given pioneering work in mammals which suggested a role for sleep in exchanging interstitial fluid and metabolites between the brain and cerebral spinal fluid (Xie et al., 2013). Recent work demonstrated that a similar function is conserved in flies via vesicular trafficking through the fly blood-brain barrier (Artiushin et al., 2018). It would be interesting to determine whether these genes function in this process.”

18. *Could the authors discuss the little overlap between males/females and shot/long sleep for 145 gene pairs identified after the MCMC runs. Similarly, how can the network differences be explained from a biological/evolutionary perspective?*

**Our response:** The reviewer asks an interesting question. We did not detect sex-specific responses to artificial selection for long or short sleep in the present experiment. Yet differences in gene expression network pairs between males and females exist, and as the reviewer mentions, we also observed differences in network pairs between long sleepers and short sleepers. These differences reflect an inescapable conclusion: a given sleep duration phenotype can originate from more than one gene expression network configuration.

19*. In the mutational analyses it is pointed out that CG12560 and Jon65Aii only affect females significantly. However, in the following sentence, the authors claim these two genes had the greatest effect on both sexes, which seems contradictory, at least in the way it is described.*

**Our response:** Our wording may have been confusing, given that it came after a comment about Jon65Aii. Our exact statement was “Effects of the Minos insertions on night sleep duration were stronger in females than in males; when sexes were examined separately, only mutations in CG12560 and Jon65Aii affected male night sleep duration.” This was meant to convey that the effects of all Minos insertions were the same directionally for both males and females, but that only CG12560 and Jon65Aii insertions had statistically significant effects on each sex separately. We have re-worded this sentence to read “All Minos insertions had the same directional effect on night sleep for both males and females, but only the CG12560 and Jon65Aii insertions had statistically significant effects on night sleep on each sex separately.”

20*. Maybe a small comment on how unchanged expression could lead to the observed phenotypical variation could help understanding how Minos mutations effects are biological mediated for those not familiar with the method. This seems to be the authors expectation so, could it be non-functional proteins or something else?*

**Our response:** The reviewer raises an interesting point. We did not observe changes in gene expression for CG13793, Cyp6a16, or hiw compared to w^1118^ controls. Thus far, we have examined gene expression relative to the control for a single timepoint, and only in pooled whole flies. Differential gene expression between the Minos mutants and controls might occur at a different timepoint, or in a small set of key neurons that would be undetectable when comparing whole flies.

We expand on this in Results, under “Mutational analyses confirms the role of candidate genes and interacting expression networks in sleep”: “Potential reasons for the lack of a significant change in gene expression in the remaining lines include: the position of the insertion within the targeted gene, which has variable effects on its expression; the relatively low statistical power of the experiment; confining our observation to a single timepoint during the day; or pooling whole flies, which might obscure gene expression changes occurring at a single-tissue level.”

21. *The assumption that interacting genes would have their expression ratio changed by the Minos insertion would hold on situation where the affected gene causally interferes with the candidates expression. As far as I understand, causality cannot be inferred by the proposed method. Thus in a situation where both genes are co-regulated by a third factor, no change in expression ratio is to expected. How would the authors re-interpret their final result when considering this direct vs indirect interaction distinction?*

**Our response:** Our method only gives us the hypothesis that two genes interact based on their correlation, and that is what we test using the Minos insertions. We do not as yet have a way to identify a third gene or factor that might be regulating the two. Given the number of genes affecting sleep, it is quite likely that there are such factors, but we can only report and test what we’ve observed. Any interpretation based on an arbitrary third factor would be purely speculative.

**Referees cross-commenting**

22*. I agree with Reviewer #2 comments which, to me, reads as generally pointing out the lack of biological interpretation of the results (and thus connecting this study with previous literature). Adding this component would make the manuscript well-rounded and attractive to a wider audience.*

**Our response:** We agree with both reviewers that additional biological interpretation of the results would make the manuscript more attractive to a wider audience. Accordingly, we have added the following paragraph to the Discussion: “The genes we identify herein overlap and extend previous work. Of the 1,140 genes implicated in the generalized linear model, 151 (13.2 percent) overlapped with previous candidate gene, random mutagenesis, gene expression, and genome-wide association studies of sleep and circadian behavior in flies (Pegoraro e t al., 2022; Dissel et al., 2015; Seugnet et al., 2017; Shalaby et al., 2018; Thimgan et al., 2010, Thimgan et al., 2018, He et al., 2013; Mallon et al., 2014; Roessingh et al., 2019, Feng et al., 2018; Lee et al., 2021; Khoury et al., 2020; Wu et al., 2018; Harbison et al., 2013; Harbison et al., 2009; Harbison et al., 2017; Harbison et al., 2019). Notably, previous studies identified the genes CG17574, cry, dro, mip120, Mtk, NPFR1, pdgy, PGRP-LC, Shal, and vari as affecting sleep duration (Feng e t al., 2018, Dissel et al., 2015; Pegoraro et al., 2022; Thimgan et al., 2018; Mallon et al., 2014; He et al., 2013; Khoury et al., 2020; Harbison et al., 2013). Two genes, ringer and mip120, overlapped with our previous study of DNA sequence variation in flies selected for long and short sleep (Harbison et al., 2017). In that study we identified a polymorphism in an intron of ringer that changed in allele frequency with selection, with increases in the population frequency of the ‘G’ allele with increasing sleep, while the frequency of the ‘A’ allele increased with decreasing sleep. When the selective breeding procedure was relaxed, the frequency of the ‘G’ allele increased in short-sleeping populations, paralleling an increase in sleep (Souto-Maior et al., 2020). One possibility is that this polymorphism contributes to the changes in gene expression in ringer that we observed in the present study. Of the 85 genes common to both sexes that we used in the gene interaction networks, 11 (13 percent) appear in other studies of sleep: CG10444, CG2003, CG5142, CG6785, CG9114, CG9676, CR42646, hiw, NPFR1, Tie, and wb (He et al., 2013; Seugnet et al., 2017; Wu et al., 2018; Harbison et al., 2013). Thus, our study corroborates genes known to affect sleep, and identifies new candidate genes for sleep as well.”

Reviewer #1 (Significance (Required)):

*This study proposes the application of advanced non-linear methods to study complex traits such as sleep. As implemented, Gaussian Processes are able to identify non-linear correlations between two biological features (e.g. transcripts) over time (e.g. generations), representing an attempt to push the analytical methods available beyond the single gene paradigm. As such, more than the relevance of the biological results themselves, the authors focus on the explaining and illustrating the application of methodological advances obtained, and its relevance to obtain a better understanding of biological systems.

However the mathematical principles required to understand the implemented method are not trivial and require advanced knowledge of machine learning and statistics. This is a potential barrier, though not an impediment, to its quick and wide adoption by the community. In addition, even if demonstrated to be a valid method when working with Drosophila, the resolution required to perform such a study may be difficult to obtain with other model systems, which would likely require further refinement of the statistical approach.

The main audience interested in this work would be basic sleep researchers. However, this work is also related to the understanding gene selection over an artificial evolutionary process, thus evolutionary and developmental biologist may be also be interested. The methodology itself, already used in other fields of study, is a general statistical tool that could be adopted by a broad range of researchers for a diversity of topics. As such, I believe with this work, the authors will be able to stimulate the development and/or rethinking of the available analytical methods to study complex biological systems, though this would likely be done either in collaboration with the authors themselves or by a specific subset of researchers who regularly work with advanced mathematical, statistical and computational principles.

(disclaimer) My mathematical formation does not reach the PhD level expertise that may be required to fully understand the methodology described. I have never personally worked with D. melonogaster or used Gaussian Processes in a professional setting. As such, I may not be able to fully evaluate/appreciate the more detailed technical aspects of this work.


Reviewer #2 (Evidence, reproducibility and clarity (Required)):

Souto-Mairo et al. reports phenotypic and genotypic effects of artificially selecting for short and long sleep in flies. They generated an impressive time-series dataset where one could examine genetic and phenotypic changes across time (generations, total 13 generations) in response to the selection pressure. The authors explored the relationships between pairs of genes in addition to just identifying potential candidate genes involved in the regulation of the amount of sleep.

Major points:*

1. *Harbison et al 2017: This study seems to be a continuation of Harbison et al 2017. There needs to be a clearer approach in the text (introduction?) in elucidating how this study is really advancing the findings of Harbison et al., 2017. Do the two studies use the same selection lines? If not, how are they different? If they are not different, what might cause the phenotypes evolving differently? For example, day sleep, day bout number do not respond to the selection pressure similarly in both studies etc.*

**Our response:** We would like to emphasize that this study is not a continuation of the Harbison et al., PLoS Genetics, 2017 paper, where we examined the changes in DNA sequence during artificial selection, and it does not use the same selection lines. The fact that the two studies are different can be seen from an examination of Figure 1A of the current study and Figure 1A of the Harbison et al 2017 study. The trajectories of each population across generation are very different. Out of convenience, we used the same nomenclature to refer to the populations in both studies (L1, L2, S1, S2, etc.), and apologize if this is the source of the confusion. Both studies do originate from the same outbred population, however, and to get to the broader question that the reviewer is asking, should one expect to see the same correlated responses to selection for night sleep among selection lines originating from the same outbred population? The answer is no, not unless the selected trait and the responding trait have a genetic correlation of 1.0. We previously estimated the correlation between day sleep and night sleep to be between 0.29 - 0.38 and between day bout number and night sleep to be -0.05 (Harbison et al., 2013; Harbison et al. 2009). In the Harbison et al. 2017 study we noted that day sleep and day bout number had correlated responses to selection for night sleep, but neither have correlated responses in the current study. The relatively low genetic correlations between these two measures and night sleep explain why we do not see a consistent correlated response among studies.

We didn’t really elaborate on these observations in the manuscript, and so have added to the Results under “Correlated response of other sleep traits to selection for night sleep” the following: “These correlated responses concur with previous observations we made in selected populations originating from the same outbred population for night sleep and night average bout length, and night sleep and sleep latency (Harbison et al., 2017). However, unlike the previous study, we did not see a correlated response between night sleep and day sleep, and night sleep and day bout number (Harbison et al., 2017). The lack of correlated response reflects the relatively low genetic correlations these two traits have with night sleep (Harbison et al., 2013; Harbison et al., 2009).”

2. *Zeitgeber Time (ZT) for RNA collection: It is puzzling that the study reports that the RNA was collected at 12 PM. I do not understand what this information means; especially in a project where one is working with sleep. The authors might want to report ZT. Also, why a particular ZT was chosen should be discussed. These genes are potential sleep-relevant genes - hence it is not too esoteric to think that the ZT of data collection matters a lot as some of them might be cycling. To get a more appropriate picture, multiple time points of data collection might be even better. The authors seem to have ignored this crucial aspect of a clock/sleep study - time of data collection and how time of data collection might shape your findings.*

**Our response:** We agree with the reviewer that it would be better to have multiple timepoints for collection, but this is difficult to implement in practice as it would require an additional 5,280 flies per generation (4 pools of 10 flies per sex per population) for 12 timepoints as recommended by Hughes et al., JBR, 2017. We mention collection time in the Methods and Materials because we are aware of the changes in gene expression over the circadian day. 12PM is the midpoint between the start of the lights-on and lights-off period (i.e., ZT6), and was chosen arbitrarily. We have added the ZT notation to the Methods and Materials for clarity*.*

3. *Short sleeping flies: Are there reports of flies sleeping this less? "We found 2,830 interactions; 8 of these were one of the 3,570 between the 85 genes, but none of them overlapped with the 145 gene pairs found to be different from controls. The gene interactions we observed may therefore be unique to extreme sleep." What is extreme sleep? How does this study then claim to have identified evolution of potential sleep-relevant gene expression for normal, physiologically relevant sleep?*

**Our response:** Our statement was not very well worded, and we thank the reviewer for noticing this. What we intended to say was that the lack of overlap between our data and a known protein-protein interaction database may due to the interactions being unique to sleep as opposed to some other complex trait. We have re-worded this statement to say “The gene interactions we observed may therefore be unique to sleep.”

*Minor points:*

4. *The article uses an unnecessarily defensive tone to establish their approach to understand underlying mechanisms of sleep 'better' than that of the others (in both introduction and discussion): "In spite the large amount of studies and data generated for many systems, identifying underlying processes is still very rare; this is clear indication that better methods are needed to obtain understanding of biological processes from data." The 'still very rare' part is just factually incorrect and misleading as far as sleep is concerned. Even if we just see Drosophila studies on sleep, there is a huge progress that's being made in terms of genes, neurons and circuits relevant for sleep: both in terms of baseline sleep as an output of the* *circadian clock and the rebound/homeostatic sleep. Most, if not all, of these elegant and pioneering studies from multiple, independent groups took approaches that did not require artificial selection regimes. As a substitution for their defense, the authors might attempt to present their findings in the context of the existing knowledge of sleep in flies. For example, what about genes already implicated in sleep? Do they show up in their analysis? For example, Sleepless, DATfmn, Sandman, AstA, AstA-receptor, Wide-awake etc. This could really help the manuscript.*

**Our response:** We certainly did not intend for this statement to suggest that no progress had been made in the identification of genes and circuits for sleep, and we agree that elegant and pioneering approaches have made significant progress in our understanding of the phenomenon. Rather, we were thinking more in terms of fully described biochemical networks. To avoid this interpretation by other readers, we have altered the “still very rare” sentence in the Introduction to read: “Despite the large amount of studies and data generated for many systems, a full understanding of underlying processes has not yet been achieved…’

We also agree with the reviewer that it would be helpful to put our work in the context of what is already known in flies. We have added the following paragraph to the Discussion to relate the work with previous work on sleep in flies: “The genes we identify herein overlap and extend previous work. Of the 1,140 genes implicated in the generalized linear model, 151 (13.2 percent) overlapped with previous candidate gene, random mutagenesis, gene expression, and genome-wide association studies of sleep and circadian behavior in flies (Pegoraro e t al., 2022; Dissel et al., 2015; Seugnet et al., 2017; Shalaby et al., 2018; Thimgan et al., 2010, Thimgan et al., 2018, He et al., 2013; Mallon et al., 2014; Roessingh et al., 2019, Feng et al., 2018; Lee et al., 2021; Khoury et al., 2020; Wu et al., 2018; Harbison et al., 2013; Harbison et al., 2009; Harbison et al., 2017; Harbison et al., 2019). Notably, previous studies identified the genes CG17574, cry, dro, mip120, Mtk, NPFR1, pdgy, PGRP-LC, Shal, and vari as affecting sleep duration (Feng e t al., 2018, Dissel et al., 2015; Pegoraro et al., 2022; Thimgan et al., 2018; Mallon et al., 2014; He et al., 2013; Khoury et al., 2020; Harbison et al., 2013). Two genes, ringer and mip120, overlapped with our previous study of DNA sequence variation in flies selected for long and short sleep (Harbison et al., 2017). In that study we identified a polymorphism in an intron of ringer that changed in allele frequency with selection, with increases in the population frequency of the ‘G’ allele with increasing sleep, while the frequency of the ‘A’ allele increased with decreasing sleep. When the selective breeding procedure was relaxed, the frequency of the ‘G’ allele increased in short-sleeping populations, paralleling an increase in sleep (Souto-Maior et al., 2020). One possibility is that this polymorphism contributes to the changes in gene expression in ringer that we observed in the present study. Of the 85 genes common to both sexes that we used in the gene interaction networks, 11 (13 percent) appear in other studies of sleep: CG10444, CG2003, CG5142, CG6785, CG9114, CG9676, CR42646, hiw, NPFR1, Tie, and wb (He et al., 2013; Seugnet et al., 2017; Wu et al., 2018; Harbison et al., 2013). Thus, our study corroborates genes known to affect sleep, and identifies new candidate genes for sleep as well.”


Reviewer #2 (Significance (Required)):

5*. I believe that the authors should attempt to put this study in the context of what is already known in sleep in flies and how this study advances the knowledge. And how the knowledge generated by this study would help other sleep researchers, who, for obvious reasons, would like to employ techniques other than artificial selection and big data.
The data is elegant. The work seems to be extremely laborious. Nonetheless, as it stands now, this manuscript is only very specific for an audience who work with artificial selection to understand underlying genetics of behavior. In fact, even within the fly sleep field, most people might not find this manuscript very useful.*

**Our response:** The reviewer may not have considered the wider application of this work. This framework is applicable to any data set of gene expression sampled across time, whether sampled across generation, as we did, or across the 24-hour circadian day, or sampled at other time intervals. We have added a statement to the Discussion to stress this fact: “The Gaussian Processes we apply herein have broad applications to other experimental designs, such as gene expression measured at varying time intervals over the circadian day, or time-based sampling of gene expression responses to drug administration.”
