# Supplementary material for: Nonlinear expression patterns and multiple shifts in gene network interactions underlie robust phenotypic change in Drosophila melanogaster selected for night sleep duration
Source: PLoS Comput Biol. 2023 Aug 10;19(8):e1011389. doi: 10.1371/journal.pcbi.1011389 (PMC10443883; doi:10.1371/journal.pcbi.1011389)
Supplement: Attachment — Submitted filename: Response to PLoS Comp Biol Reviews 051823.docx [file pcbi.1011389.s026.docx]

We thank the reviewers for their careful evaluation of our manuscript. Several important points were raised by the reviewers, and we have addressed them, improving the manuscript. Reviewer comments are in italics; our responses are provided in blue plain text.

Please note that the original manuscript was provided as a LaTeX-generated .pdf; we have made tracked changes to this .pdf (marked in blue). The revised ‘clean’ copy of the manuscript was generated using the *PLoS Computational Biology* LaTeX template, so the format of the revised paper has also changed to adhere to *PLoS Computational Biology* requirements.

Reviewer #1: • What are the main claims of the paper and how significant are they for the discipline?
In this manuscript the authors developed a Gaussian Process model to address the limitation of the current methods in capturing non-linear covariance between genes over time. Given how the assumption of linearity is expected to be often violated in complex biological systems, this is a significant methodological advance into exploring and describing complex interactions bio-chemical interactions, for example, and as illustrated, to study associations of transcript-phenotype during artificial selection.

***Our response:*** We thank the reviewer for this comment!

• Are these claims novel? If not, which published articles weaken the claims of originality of this one?
Thought the statistical and mathematical principles of the methodology developed have been studied and applied in other fields, to the best of my knowledge, it is the first time such methodologies are used to explore the selection of gene networks during selective breeding for extreme sleep phenotype.

• Are the claims properly placed in the context of the previous literature? Have the authors treated the literature fairly?
This was one of the points raised by the previous reviewers and the authors have addressed it by providing a more complete discussion of the previous biological knowledge.

• Do the data and analyses fully support the claims? If not, what other evidence is required?
The data supports the authors’ claims. In addition to the computational aspect of the work, and despite it being impossible to confirm all results from high through-put computational modeling, the authors present relevant biological evidence in support of their findings.

• Would additional work improve the paper? How much better would the paper be if this work were performed and how difficult would it be to do this work?
These issue has been raised by the previous reviewers and addressed properly in my opinion.

• PLOS Computational Biology encourages authors to publish detailed protocols and algorithms as supporting information online. Do any particular methods used in the manuscript warrant such treatment? If a protocol is already provided, for example for a randomized controlled trial, are there any important deviations from it? If so, have the authors explained adequately why the deviations occurred?
The underlying mathematical principles of this work have been described, though not in sleep research literature. As such a detailed description of the algorithms behind it is not necessary.

• Are original data deposited in appropriate repositories and accession/version numbers provided for genes, proteins, mutants, diseases, etc.?
The sequencing data has been made available on the National Center for Biotechnology Information (NCBI) Gene Expression Omnibus (GEO) under the accession number GSE202600.

• Has the author-generated code that underpins the findings been made publicly available?
The code used has been made available on GitHub.

• Does the study conform to any relevant guidelines such as CONSORT, MIAME, QUORUM, STROBE, and the Fort Lauderdale agreement?
As far as I can tell the study conforms with all standards.

• Are details of the methodology sufficient to allow the experiments to be reproduced?
The information provided in the methods and online should be enough to reproduced the manuscript, though it may be difficult for non-experts to use it without a walk through or tutorial guide.

• Is any software created by the authors freely available?
Yes, all code has been made available on GitHub

• Is the manuscript well organized and written clearly enough to be accessible to non-specialists?
The manuscript is well organized and easy t follow, though non-specialists lacking the mathematical background required may not find it accessible.

• Does the paper use standardized scientific nomenclature and abbreviations? If not, are these explained at the first usage?
All nomenclature is standard or properly explained.

• Additional points.
For the figures, it could be helpful to add the color legend to the side, so one doesn’t need to read the text every time to find out which phenotype is which color.

***Our response:*** We have added color legends to Figures 1 and 6 of the main text. We also clarified the Figure 6 legend to indicate that circles refer to the *w*^1118^ control background, while triangles refer to the *y*^1^*w*^67c23^ control background.

Since it was discussed with the previous reviewers, it could be worth mentioned a little on ZT6 (‘middle of the day’) fits in the fly sleep-wake cycle, since their behavior differs from other animal models.

***Our response:*** Flies, like humans, are diurnal, i.e., they are active during the day and sleep at night. As we only sampled flies from a single time point, the choice of time was arbitrary. We clarified this in the Methods section under ‘RNA extraction and sequencing’: “This timepoint was arbitrarily chosen and is during the fly’s active period.” Please see also the response to Reviewer 2’s comment below, where we address an additional comment on the circadian cycling of gene expression.

For the section ‘Phenotypes in flies used for RNA-seq’ , I would intuitively expect that the unselected control flies would be more correlated with the population they were drawn from than the selected groups (short- and long-sleepers). This is mostly due to the fact that the selected populations are expected to be the phenotypical outliers, so the furthest away from the mean behavior. Could the authors comment or clarify these observations?

***Our response:*** As the reviewer notes, the phenotypes of flies sampled from the long- and short-sleep selected populations are better correlated with the overall population mean than the phenotypes of flies sampled from the control populations. The reason is that selective breeding for long and short sleep resulted in greater inbreeding than the randomly-bred controls, so that a given fly from a selected population is going to be more genetically similar to its cohorts than a fly from a control population. The fact that sleep phenotypes in the controls didn’t change much over time is another indicator that the control populations were not subject to much inbreeding. This is not necessarily going to be the case for all selective breeding experiments, however, which is why the control populations are so important. We have added a statement to the Results, under ‘Phenotypes in flies used for RNA-Seq’: “The lower correlations observed in control flies indicate that they were less inbred than the selected populations.”

Reviewer #2: Summary
In the manuscript entitled “Nonlinear expression patterns and multiple shifts in gene network interactions underlie robust phenotypic change in Drosophila melanogaster selected for night sleep duration”, the authors used a Gaussian Process (GP) model to systematically identify changes in interactions between genes over the generations of artificial selection. Specifically, they conducted an artificial selection experiment with Drosophila melanogaster, and, every generation, they harvested RNA from files. Then, they established a multi-channel GP model for estimation of the nonlinear expression patterns of genes related to sleep phenotypic change, which is impossible with a linear model analysis as mentioned in the manuscript. Using the GP model, they identified some genes whose expression level significantly changes in the artificial selection. Lastly, to validate part of the findings obtained using the GP model, mutational analyses were performed by a Minos insertion. These results clearly demonstrate the usefulness of the GP-based approach in investigating the complex molecular interactions leading to diverse phenotypes. I believe that the approach is interesting and correctly applied. Considering this, the current manuscript well fits the scope of the PLOS Computational Biology, and thus I recommend the publication. However, I provide major and minor comments to be solved before the publication in below.

Major comments
1. To understand this work, readers must have both experimental and mathematical/statistical backgrounds, as Reviewer 1 mentioned. However, several potential readers of this work, such as evolutionary biologists and clinician in sleep research field might not have enough mathematical/statistical backgrounds. I believe that this problem frequently occurs in interdisciplinary research. I felt that, to circumvent this problem, the authors described their methodology in detail in the Methods and Materials, which is good. However, even with the detailed description, the manuscript can lose potential readers due to the inherent difficulties of the methodology, making readers a little bit stressful. To completely avoid this circumstance, I kindly recommend that the authors provide a user-friendly computer package to implement their GP algorithm. Here, user-friendly means that users do not need to understand all the code, and, by just changing the input (e.g., RNA-seq data), they can run the code and analyze their own data. After this, it would be great if the authors clearly mention that they provide an easy-to-use and user-friendly computer package for implementation of the algorithm, for example, in the introduction section.

***Our response:*** We appreciate the reviewer’s enthusiasm for our work, but we are philosophically at odds with a portion of this comment. We do not agree that it would be a good idea to create a computer package where the “…users do not need to understand all the code, and, by just changing the input (e.g., RNA-seq data), they can run the code and analyze their own data.” If users don’t know what they are doing with their data by running a piece of code, then they shouldn’t run it. We think that anyone wanting to analyze their data in a similar manner would need to have an understanding of the math and associated code. We agree with the reviewer that there are difficulties in communication in interdisciplinary research, and we’ve attempted to make our approach and the findings clear in the manuscript. Importantly, the production of a general-purpose package for RNA-seq data was not the goal of this work. Such a package would require considerable time and effort to create (more than the 60 days given for this response), troubleshoot, and maintain, and we are not certain that the demand for such a package exists at this point. We’ve made the code and data sets available in the GitHub repository along with tutorial-like documentation so that people who are interested can go through the analysis and reproduce the results.

2. Approaches based on GP models have been recently used to infer dynamics of complex biological systems. Specifically, Yang et al. (Yang et al. PNAS 2021 PMID: 33837150) proposed a method called MAGI that use a Gaussian process model over time series data. It would be great if the authors can clearly describe advantages (or novelty) of the method proposed in the manuscript compared to the MAGI in the manuscript.

***Our response:*** It is true that the Yang *et al.* PNAS paper that the reviewer mentions applies a Gaussian process model to time series data. However, they specifically apply their method to the solution of ordinary differential equations (ODEs), including those that describe both gene regulation and circadian biology. As Yang et al. state, ODEs have the general form:

$$\frac{dx(t)}{dt}=f(x\left( t \right), \theta, t)$$

ODEs are useful for modeling dynamic systems when the structure of $f$ is *known*. They list examples that are well known and studied in the literature. These include the oscillation of Hes1 mRNA used as an example by Yang et al., which is assumed to follow Goodwin’s oscillatory model of a negative feedback loop. Hes1 mRNA fluctuations are governed by rates of its transcriptional initiation and protein translation as well as protein and transcript degradation, the concentration of Hes1 protein that will repress its transcriptional initiation, and the Hill coefficient. These biochemical parameters enable the construction of ODEs that approximate these oscillating processes, and MAGI provides a means for their rapid estimation.

As far as we are aware, the data set we collected here, sampling RNA over every generation during artificial selection in flies (one sample every 2.5 weeks, ~ 37 weeks total) is the first of its kind. The dynamic processes that would describe the change in gene expression across the transcriptome over generations of time are as yet unknown. Hence in our case the “$f$” is unknown. Moreover, we would need to know how gene expression fluctuates over generational time for every gene in the genome, i.e., an $"f"$ for every gene in the genome. Instead of attempting to model the “*f*” explicitly, we use the Gaussian Process to apply the squared exponential kernel, which effectively models an infinite number of potential basis functions, to fit the data. We therefore do not have to specify an “$f"$ to gain some information about the underlying gene expression covariance matrix.

Thus, MAGI does not apply to our data as we do not have ODEs to solve, and our method wouldn’t solve the ODEs from the MAGI paper either. The two methods address entirely different types of problems. We have added the Yang *et al*. reference in the Materials and Methods under ‘Gaussian Process regression’ and in the Discussion where we acknowledged others applying the Gaussian Process to ODEs as the omission of the Yang *et al*. reference was not intentional.

3. Because the authors said “we develop a flexible Gaussian Process model …” in the abstract, and the manuscript focuses on the application of the method to experimental data, I believe that the authors need to describe previous approaches of network analysis potentially related to the proposed GP method (e.g., Langfelder & Horvath BMC Bioinformatics 2008 PMID: 19114008; Yang et al. PNAS 2021 PMID: 33837150; Horvath & Dong PLOS Comp, Biol. 2008 PMID: 18704157) in the introduction section. Moreover, it would be great if the authors can more clearly describe the novelty of this work. Specifically, if there is a methodological advance in the proposed method compared to the previous ones, please clearly describe it. If the main contribution of the manuscript is a specific application of the previously developed GP method and identification of previously unknown biological knowledge, please more focus on describing the biological findings instead of describing the adopted methodology.

***Our response:*** We aren’t quite sure what the reviewer intends by these comments. We do mention the differences between the Langfelder and Horvath paper (which describes the WGCNA method) and our work in the S1 Appendix (also, the Horvath and Dong paper is an extension of WGCNA). As we state above, the Yang et al. paper does not solve the same type of problem that we solve in our manuscript. This work is of a multidisciplinary nature, so we cannot place all of the emphasis on either the computational methods or biological conclusions alone. The experimental  design, the statistical methodology/implementation, and the biology are inextricably linked. The choice of journal, *PLoS Computational Biology*, is also a reflection of that fact. Therefore, the original methods have given rise to novel biological insights, specifically identifying both known as well as novel genes and gene interactions. Roughly 13% of the genes we identified for sleep have been found via other approaches, as we detail in the Discussion; the remaining genes we identify are novel.

Minor comments
1. As the author described in the Methods and Materials, 10 files were chosen from 75 files and frozen for RNA extraction at ZT6. Among the extracted RNAs, levels of some RNA might oscillate over time, as the Review 2 mentioned. Thus, the conclusion of the manuscript may change depending on the collection time of RNA. It would be great if the authors discuss this point in the discussion section in addition to mentioning the collection time in the Methods and Materials. At least, it might be needed to mention that the future work that checks whether the findings are preserved even with the samples collected at different times in the manuscript.

***Our response:*** We thank the reviewer for this comment. It seems unlikely that the conclusion of our manuscript would change if the genes were shown to cycle. We compared the genes that were significantly associated with selection scheme over generation (405 genes for males; 820 genes for females; 85 genes overlapping) with genes that have been reported in the literature to have cycling gene expression in fly heads and bodies. Of the 405 genes that we identified for males, 47 (11.6%) are known to cycle. Of the 820 genes that we identified for females, 170 (20.7%) are known to cycle. Of the 85 genes overlapping between males and females, 13 (15.3%) are known to cycle. Thus, most of the genes we identified do not have cycling gene expression, and our conclusions would not change concerning these genes. For genes that do cycle, our conclusions for a given gene would change only if the gene cycled at significantly different phases among selection populations.

We have added these findings as a paragraph in the **Discussion**: “Here we extracted RNA from flies at a single circadian timepoint, ZT6. However, gene expression is known to cycle in fly heads and bodies, begging the question of whether the genes we identified at a single timepoint are subject to cycling over the 24-hour day. We therefore compared our list of genes that were significantly associated with selection scheme over generation (405 genes for males; 820 genes for females; 85 genes overlapping both sexes) with genes known to have cycling expression (McDonald and Rosbash, Cell, 2001; Ceriani et al., J. Neurosci, 2002; Lin et al., PNAS, 2002; Ueda et al., J Biol Chem, 2002; Hughes et al., Genome Res, 2012; Rodriguez et al., PNAS, 2013; Kumar et al., J Biol Rhythms, 2020). We found that 47 of the 405 genes identified for males cycle (11.6%), 170 of the 820 genes identified for females cycle (20.7%), and 13 of the 85 genes overlapping between males and females cycle (15.3%). Thus, most of the genes we identified are not known to cycle over the 24-hour day.”

2. I could not access the Git Hub repository via the link provided in the Data Availability section. It would be great if the authors can check whether the link works.

***Our response:*** The link we provided is correct: <https://github.com/caesoma/Multiple-shifts-in-gene-network-interactions-shape-phenotypes-of-Drosophila-melanogaster>

However, the link in the draft manuscript will not work if one simply clicks on it because it will include the line number from the draft version of the manuscript. We apologize for the difficulty.

3. I believe that this technique can be applied to other biological systems, such as gene regulatory networks associated with Fatty acid Composition (Basnet et al. Plant Physiol. 2016 PMID: 26518343) and cell signaling networks associated with antibiotic stress (Kim et al. Science Advances 2022 PMID: 35302852). It would be great if the authors mention this point in the discussion section.

***Our response:*** We are not sure whether we completely understand the reviewer’s comment. We have no doubt that the technique can be applied to other biological systems, and stated as much in the Discussion. However, we wouldn’t dream of singling out specific works to suggest that they could be somehow improved if only the authors applied our method to their data. It is up to the authors of those works to determine whether our method would be beneficial to them.
