# Supplementary material for: Nonlinear expression patterns and multiple shifts in gene network interactions underlie robust phenotypic change in Drosophila melanogaster selected for night sleep duration
Source: PLoS Comput Biol. 2023 Aug 10;19(8):e1011389. doi: 10.1371/journal.pcbi.1011389 (PMC10443883; doi:10.1371/journal.pcbi.1011389)
Supplement: Attachment — Submitted filename: Response to PLoS Comp Biol 2nd Reviews Fin.docx [file pcbi.1011389.s027.docx]

Reviewer #2: I appreciate the thorough response of the authors to my comments, which effectively addressed several of my concerns. Nevertheless, there are still one remaining concern that should be addressed before the manuscript can be published in PLoS Computational Biology. See below for details.

Concern 1 (regarding the third major comment):
The authors developed a novel GP model for analyzing nonlinear expression patterns in gene network interactions and applied it to biological data. I acknowledge their methodological advancement in this regard. However, it is important to ensure that the authors have clearly described their approach in relation to existing literature and have treated the literature fairly, as emphasized by previous reviewers.

Typically, when introducing a new methodological advancement in a manuscript, it is necessary to carefully discuss and contextualize previous methods in the introduction and discussion sections. The authors have made improvements to the discussion section based on my comments about MAGI, which is commendable. However, they have not adequately introduced any previous methods in the introduction section. It appears that the authors believe that mentioning the previous literature about GP models in the supplementary information (i.e., appendix) is sufficient. I am not sure whether this approach is enough to treat the previous literature fairly, especially considering that one of the main contributions of the manuscript is the development of a new GP model.

To the best of my knowledge, when the authors introduced their methodology in the introduction section, they merely stated, "The RNA sequence data, which consisted of expression levels as a function of time (measured in generations), was analyzed using a Multi-Channel Gaussian Process [37, 38]." I am unsure if this single sentence adequately conveys to readers that the authors have developed a new method distinct from previous approaches in the manuscript.

In conclusion, I believe that the findings presented by the authors meet the high-quality standards required for publication in PLoS Computational Biology. However, significant improvements are needed in the writing to address the aforementioned concerns.

***Our response:*** Since the reviewer notes references 37 (Melkumyan and Ramos, 2011) and 38 (Bonilla et al., 2008) in particular, we would like to point out that these papers already have a prominent place in the Methods and not just in the Appendix. Under “Multi-channel Gaussian Processes”, we stated: “The different dependent variables yi are sometimes called a multi-task or multi-channel Gaussian Process. The details of the specification of this model can be found in 38 and 37, which we summarize below.” We then go on to describe the multivariate gaussian with signal and covariance matrices, referencing both papers again in the next 16 lines (lines 265-284 in Methods).

We certainly did not intend to confine any particular reference to the Appendix. We have ensured each reference in the appendix also appears in the main text. In the Introduction, we added the references to MMC and WCGNa to Line 65: “Correlation (Langfelder and Horvath, 2008; Stone and Ayroles, 2009) or information-based theory methods…”

In addition, we have added an additional paragraph on Gaussian Processes in the Introduction that supplies the context for our work:

“Recent work applies Gaussian Process models (Rasmussen and Williams, 2006; Schulz *et al*., 2018) to data sampled over time in order to evaluate dynamic parameters. Where gene and protein expression dynamics can be modeled with differential equations, Gaussian Process methods estimate parameters for non-linear systems (Yang *et al*., 2021), elucidate the spatial-temporal dynamics of developmental morphogen gradients (Liu and Niranjan, 2011), model signaling and gene regulatory networks (Aijo *et al*., 2013; Aalto *et al*., 2020), infer latent transcription factor activity (Gao *et al*., 2008), and find transcription factor targets (Honkela *et al*., 2010). Other applications of Gaussian Processes account for missing or irregularly sampled gene expression data (Hensman *et al*., 2013), model spatial interactions between cells (Arnol et al., 2019), and generate clusters (Hensman *et al*., 2013; McDowell *et al*., 2018). Gaussian Processes can also infer relationships among multiple disparate data types (Melkumyan and Ramos, 2011; Bonilla *et al*., 2008) to explore, for example latent processes underlying spatial-temporal relationships among brain structure, brain activity metrics, and behavior (Bahg *et al*., 2020), or relationships among multi-modal spatially- and temporally-varying data (Velten *et al*., 2022). Gaussian Processes thus provide a flexible framework for the estimation of latent relationships.”
